# Degradation dynamics of lead-halide perovskites under combined heat and light

**DOI:** 10.1039/d6ee04175b

**Published:** 2026-09-17

**Authors:** Alan R. Bowman, Qimu Yuan, Joshua R. S. Lilly, Adam T. M. Wood, Vincent J.-Y. Lim, Michael B. Johnston, Nakita K. Noel, Laura M. Herz

**Affiliations:** a Department of Physics, University of Oxford, Clarendon Laboratory Parks Road Oxford OX1 3PU UK laura.herz@physics.ox.ac.uk

## Abstract

Reliable predictions of long-term stability are essential as halide perovskite solar cells approach widespread commercial deployment. However, extrapolation from aging tests requires knowledge of material degradation dynamics, which remain unclear for halide perovskites. Here, we reveal the structural and optoelectronic evolution of formamidinium–caesium lead-halide perovskite films under heat and/or light using *in situ* X-ray diffraction and photoluminescence. Heat and light combined induce much faster degradation than either stressor alone, with 1 sun illumination degrading material 53 times faster at 85 °C than room temperature. For all compositions investigated the degradation rate is highly nonlinear in time and follows a sigmoidal decay, suggestive of interface-driven dynamics. Degradation proceeds in three steps: halide segregation (for mixed-halide perovskites), A-site cation diffusion triggered by heterogeneity and strain, and finally iodine and formamidinium loss. Our findings provide a quantitative framework for lifetime predictions and highlight the necessity of combined heat and light ageing tests.

## Abstract 2

The photothermal degradation dynamics of device-relevant halide perovskites is revealed through *in situ* photoluminescence and X-ray diffraction. Degradation is highly nonlinear with time and proceeds 53 times faster at 85 °C than at 22 °C.

## Graphical abstract

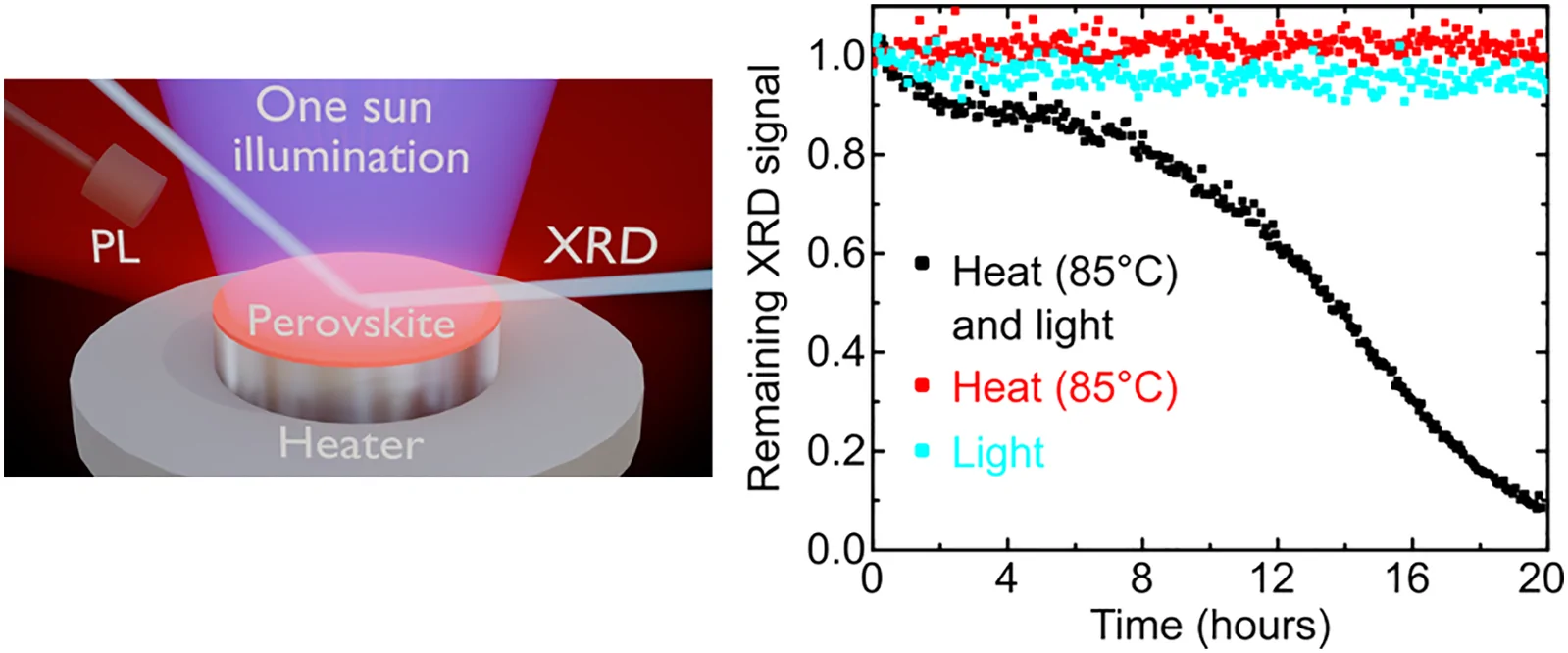

**Broader context**

Metal halide perovskites are among the most promising materials for next-generation solar cells offering high efficiencies and low manufacturing costs. A major barrier to widespread commercial deployment is uncertainty regarding long-term operational stability. To address these issues, accelerated ageing tests are increasingly being used, in which long-term device lifetimes at operating temperatures are extrapolated from tests conducted at elevated temperatures over a shorter time period. However, reliable predictions require a complete understanding of the degradation dynamics and acceleration factors. Current accelerated ageing approaches often assume degradation rates remain constant with time, yet this assumption has rarely been tested directly at the material level. Here we use multi-modal spectroscopy to record structural and optoelectronic degradation dynamics *in situ* over time. Degradation proceeds significantly faster under combined light and heat than for either stressor alone. Importantly, we find the intrinsic material degradation rate is nonlinear and initially accelerates with time. This underlines that linear extrapolation may not be a suitable assumption for degradation of halide perovskites. Overall, degradation proceeds through three phases: halide segregation; cation diffusion; and molecular loss. This work provides a complete picture of degradation dynamics, enabling reliable accelerated testing of photovoltaic devices towards the commercial deployment of solar cells.

## Introduction

Halide perovskites are highly promising candidate materials for next-generation solar cells, either alone or when combined with silicon in tandem solar cells. Certified research cell efficiencies are currently at 28.0% and 35.2% respectively for these device architectures.^1^ The main bottleneck preventing widespread adoption of halide perovskite solar cells is stability concerns, resulting in uncertainty over whether devices can operate for 20–25 years.^2^ Addressing these challenges requires protocols that can reliably predict long-term performance from aging tests under a variety of stressors, including heat, light and humidity. However, the dynamics of halide perovskite material degradation remain unclear, limiting confidence in device stability predictions. For example, device degradation rates are frequently assumed constant in time, but it is unclear whether this is the best-case scenario for halide perovskites.^3–5^

Halide perovskites have the chemical formula ABX_3_, where the A-site is typically occupied by methylammonium (MA), formamidinium (FA) or Cs; the B-site by Pb or Sn; and the X site by a halide (I, Br or Cl), or alloyed combinations thereof. The degradation products and mechanisms of halide perovskite films have been explored under a variety of stressors including humidity,^6–8^ oxygen,^9,10^ vacuum,^11,12^ light,^13^ heat^14,15^ or a combination thereof.^16,17^ Generally, these studies demonstrate breakdown of the perovskite structure, the formation of degradation compounds including Pb and PbI_2_ (or hydrated compounds when water is present *e.g.* PbIOH) and outgassing of I_2_. MA has typically been found to be the least stable of the possible A-site cations, resulting in the alternative use of mixed FA-Cs compositions in solar cells^18,19^ with mixing ratios chosen to satisfy the Goldschmidt tolerance factor. However, such mixed A-site occupation has resulted in A-site cation segregation emerging as an additional source of instability.^20–24^ In addition, device-scale degradation studies have revealed delamination and ion migration between layers as further degradation mechanisms.^25–29^ Further details on metal halide perovskite material and device degradation can be found in several review articles.^30,31^

To predict long-term stability from accelerated ageing tests, the lifetime of photovoltaic devices at room (or operating) temperature has been extrapolated from their performance at high temperature.^3,4,24^ Zhao *et al.* initially implemented this approach for halide perovskites using a model proposed by Haillant *et al.*^5,32^ Here it is assumed that the device degradation rate follows an Arrhenius-type model, allowing for the calculation of a degradation acceleration factor between room and elevated temperature. As noted in Haillant *et al.*'s original work, for this approach to be fully reliable, the degradation rate must be constant with time at any given temperature, an assumption whose validity has been uncertain to date. Additional studies have also found strong correlation between elevated temperature degradation and real-world device performance.^33^

Importantly, degradation of FA-based halide perovskites has mainly been investigated through *ex situ* characterisation post stressing. While such studies have provided useful information on the degradation products, they cannot confirm the veracity of accelerated ageing protocols. *Ex situ* studies do not inform on how materials evolve with time, so it is unclear whether degradation is linear or sudden. Device-scale studies do record current and/or voltage *in situ* over time, but may be influenced by use of specific architectures that are constantly evolving while being optimised. Therefore, degradation rates extracted from photovoltaic device performance may not reflect the best scenario available for a given perovskite absorber material. An important first step in resolving the degradation dynamics of the halide perovskite material was presented by Tang *et al.*^34^ Here, MAPbI_3_ was exposed to 77 °C with and without light. Light was demonstrated to accelerate degradation compared to heat alone *via in situ* X-ray diffraction (XRD). However, perovskite degradation was extremely rapid meaning limited information on degradation rates was obtained, and no *in situ* optoelectronic information was recorded. Further, it was unclear whether the main driver of degradation is light alone, or heat and light combined as no room-temperature measurements were taken. To the best of our knowledge, to date, no study has revealed the intrinsic structural and optoelectronic photothermal degradation dynamics of FA/Cs halide perovskites.

Here we present the degradation dynamics of PMMA-encapsulated thin films of lead-halide perovskites under the stressors of light, heat, and both together *via in situ* characterisation, concurrently revealing changes in structural and optoelectronic properties. Our approach is based around a custom-built insert to a standard X-ray diffractometer and allows for the fundamental degradation processes of the halide perovskite absorber layer to be revealed. This methodology complements studies reporting performance degradation of full photovoltaic devices, with the understanding gained allowing for suitable strategies to be developed to enhance stability. As exposure of halide perovskites to humidity, oxygen and vacuum can be partially prevented by encapsulation,^35,36^ we focus on the unavoidable effects of light and heat exposure. We study FA_0.83_Cs_0.17_Pb(I_0.7_Br_0.3_)_3_ and FA_0.83_Cs_0.17_PbI_3_ films, comparable compositions to materials used in the highest efficiency single-junction and perovskite/Si tandem solar cells.^37–41^ A mixture of FA and Cs is used in the A-site to ensure formation of a stable cubic phase at both room temperature and 85 °C.^42–45^ FA_0.83_Cs_0.17_Pb(I_0.7_Br_0.3_)_3_ is specifically chosen as it has an ideal bandgap for tandem solar cells of halide perovskite on silicon. However, it also has issues associated with halide segregation, where bromide and iodide form sub-domains when the material is illuminated.^46–48^ FA_0.83_Cs_0.17_PbI_3_ represents a promising material for single junction solar cells that does not undergo halide segregation. We used no passivation agent in thin-film fabrication, and note that such treatments may affect degradation timelines compared to passivated films.^4,37^

We reveal that heat and light cause significantly faster degradation than either stressor alone. We demonstrate that this photothermal degradation is a highly nonlinear process, which first proceeds slowly, but then accelerates as the material becomes more degraded, before slowing again once most material has degraded. Thus, a previously proposed model based on time-invariant degradation rates^5^ may not be sufficient to predict the long-term stability of devices. We show that instead the degradation follows a sigmoidal decay curve, suggesting interface-driven dynamics. We find degradation is governed by redistribution of A-site cations in all perovskites investigated, and notably we do not observe the formation of Pb or PbI_2_ degradation products here (though these are likely present as small amorphous structures). Our results underline the need to suppress A-site cation mobility for stable solar cell operation.^49^ Finally, we reveal that for FA_0.83_Cs_0.17_PbI_3_ heat (85 °C) accelerates degradation processes under light by approximately a factor of 53, compared to illumination at ambient temperature (22 °C). Overall, our study provides vital information on the intrinsic mechanisms of halide perovskite degradation and highlights that future ageing tests should prioritise photothermal stressing.

## Results and discussion

To probe perovskite degradation we applied elevated heat (85 °C) and/or light to a sample held within an X-ray diffractometer while simultaneously recording photoluminescence and thermal emission, advancing an approach our group has pioneered over the last five years.^50–53^ Perovskite films of ∼500 nm thickness were deposited from solution onto quartz substrates and encapsulated with a ∼100 nm thick layer of polymethyl methacrylate (PMMA) to prevent direct moisture or air exposure (see Methods section below for details of fabrication). This represents a comparable situation to encapsulant approaches used in perovskite stability research,^36^ with further discussion on the choice of PMMA provided in SI, Note S1. Samples were mounted on a heating stage and excited by a quasi-monochromatic light emitting diode (LED) with intensity set to generate 1 sun equivalent illumination (see Methods). We used an LED wavelength of 470 nm in all measurements unless otherwise stated, and we note that due to rapid charge diffusion in halide perovskites, charges will be distributed quite uniformly across the film depth profile independent of excitation wavelength.^54^ We employed additional temperature monitoring through a thermal imaging camera and saw no measurable change in sample temperature following LED illumination (Fig. S1). Single-wavelength excitation allows us to monitor photoluminescence, probing optoelectronic properties, while X-ray diffraction (XRD) yields information on dynamic changes in the crystal structure. A two-dimensional X-ray detector was used to monitor the 2*θ* range of 10.4°–16.1°, which includes the most intense halide perovskite peak (between 13.9° and 14.5°) and lead iodide (at ∼12.7°). We also recorded signal from 2*θ* = 27.0–33.6° every 40 minutes, allowing us to check for the formation of metallic lead.^34^ We note that such a study of thin films corresponds to the device-equivalent of open-circuit voltage, which is a harsher device testing condition than being held at maximum power point.^55^ A setup schematic is presented in the inset of Fig. 1a.

**Fig. 1:**
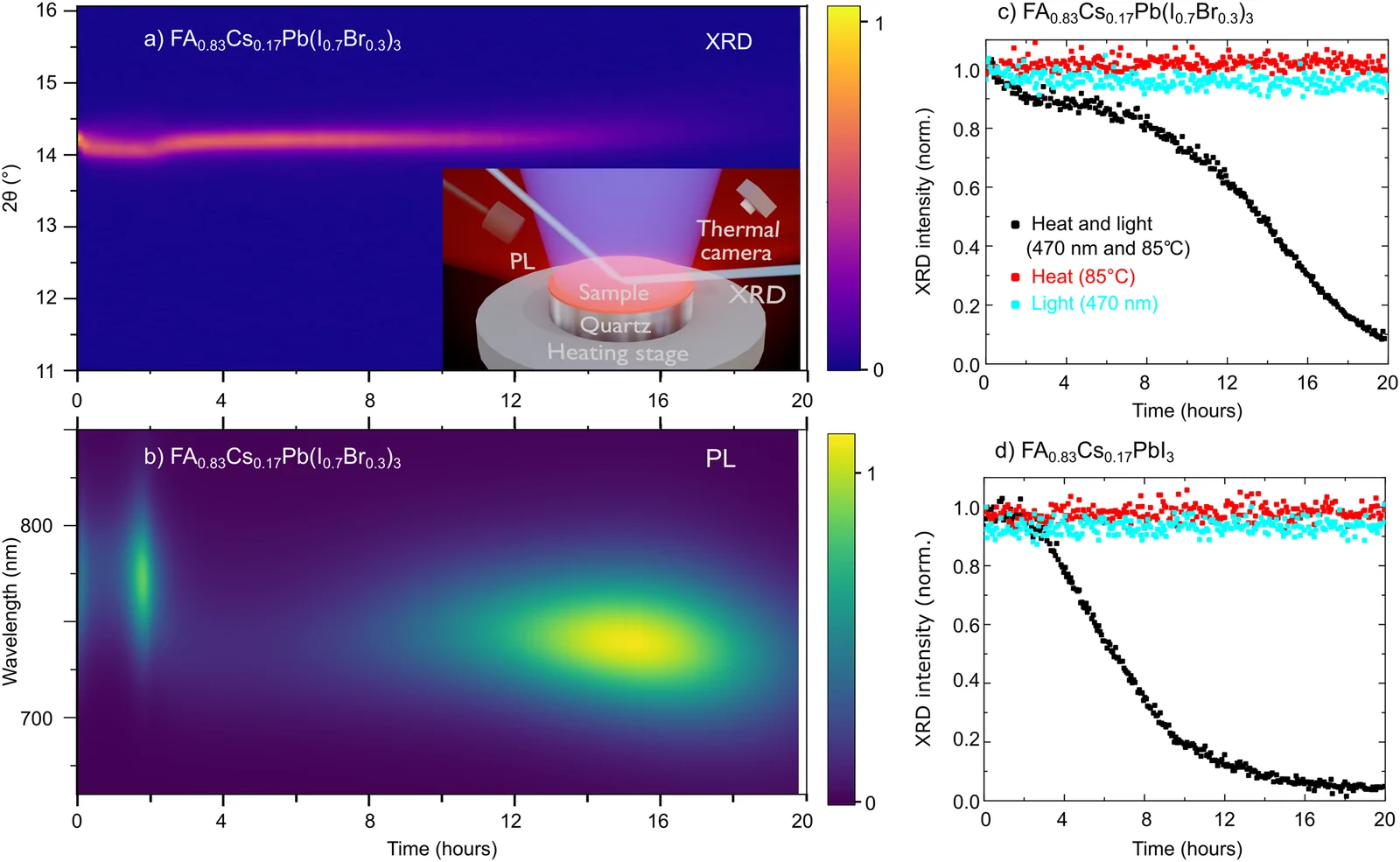
Metal halide perovskite degradation under heat, light, and combined heat and light. Normalised (a) X-ray diffraction and (b) photoluminescence signal from FA_0.83_Cs_0.17_Pb(I_0.7_Br_0.3_)_3_ as a function of time for which the sample was exposed to 470 nm (1 sun equivalent) illumination and 85 °C heat. Total XRD signal about the halide perovskite (100) peak (see Methods) normalised to initial signal as a function of time during which (c) FA_0.83_Cs_0.17_Pb(I_0.7_Br_0.3_)_3_ and (d) FA_0.83_Cs_0.17_PbI_3_ are exposed to 470 nm illumination and 85 °C, or only one of these stressors. Legend in (c) applies to both (c) and (d).

To reveal device-relevant degradation, we used conditions defined by the consensus stability tests for halide perovskite photovoltaics.^56,57^ We focused on the light-soaking (ISOS-L) and light-cycling (ISOS-LC) protocols, which are carried out at room temperature, 65 °C and 85 °C with 1 sun illumination. We present an example of the XRD and PL data obtained from our setup for FA_0.83_Cs_0.17_Pb(I_0.7_Br_0.3_)_3_ films exposed to 20 hours of continuous 85 °C and 1 sun equivalent illumination in Fig. 1a and b respectively. The XRD signal rapidly shifts to shorter angles at early times, returns to approximately its initial 2*θ* position after ∼2.5 hours and then decreases in intensity over the rest of the scan (Fig. 1a and c). The PL intensity fluctuates over the measurement window and dramatically shifts to shorter wavelengths at long times (Fig. 1b). We explore the intricacies of such data below, but in the first instance, these observations highlight that structural and optoelectronic data give complex complementary information on perovskite degradation, allowing a complete mechanistic picture to be formed.^52^

We first explored the role of heat, light or both stresses combined to understand the evolution of degradation under different accelerated testing conditions. We present the total XRD intensity of the (100) perovskite peak (indicating the remaining crystalline material) for FA_0.83_Cs_0.17_Pb(I_0.7_Br_0.3_)_3_ and FA_0.83_Cs_0.17_PbI_3_ under each of these stresses in Fig. 1c and d respectively. XRD intensity decreases significantly when these perovskite films are exposed to heat and light over 20 hours (fitting of these data is discussed in the context of Fig. 2). Conversely, for films exposed only to either light or heat, XRD intensity remains mostly flat, and within 10% of the initial value. We repeated these measurements on several fresh samples and always found comparable results (see SI, Note S3). We note that this is in contrast to what is observed for silicon solar cells, for which the addition of light increases degradation minimally beyond that recorded at elevated temperature alone.^58,59^ However, as we show in detail further below, the addition of elevated heat on its own causes a larger acceleration in degradation reported for silicon^29,60,61^ than we observe here for metal-halide perovskites.

**Fig. 2:**
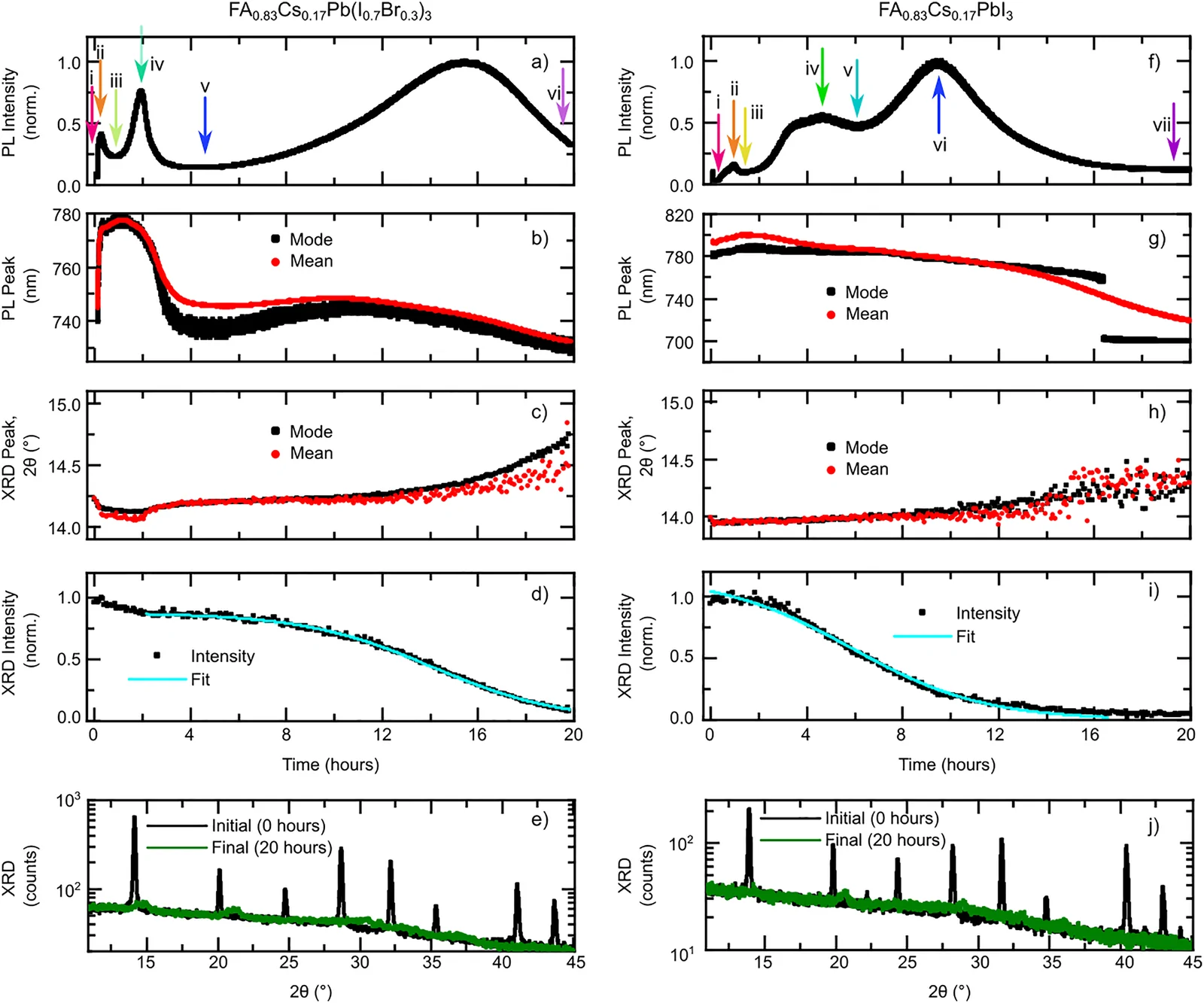
Perovskite degradation dynamics revealed by *in situ* PL and XRD. (a) Photoluminescence intensity, (b) photoluminescence peak wavelength, (c) XRD peak angle and (d) XRD intensity as a function of time for a FA_0.83_Cs_0.17_Pb(I_0.7_Br_0.3_)_3_ film during exposure to 85 °C and 470 nm illumination normalised to initial signal. Here mode describes the peak position and mean the weighted average. (e) Initial and final room-temperature XRD pattern. Equivalent data for FA_0.83_Cs_0.17_PbI_3_ is shown in (f)–(j). The arrows and roman numerals in (a) and (f) correspond to points in time for which PL and XRD data are shown in Fig. 3.

We explored how varying photothermal conditions affected the degradation pathways introduced. We heated FA_0.83_Cs_0.17_Pb(I_0.7_Br_0.3_)_3_ to 65 °C rather than 85 °C (Fig. S7) and exposed FA_0.83_Cs_0.17_Pb(I_0.7_Br_0.3_)_3_ to 590 nm illumination rather than 470 nm (*i.e.* below the bandgap of PbI_2_ to rule out its excitation driving degradation, Fig. S8). In both cases we obtained comparable results, with lower temperature resulting in a slower degradation rate. We also obtained similar results when cycling 12 hours of photothermal stressing (12 h on, 12 h off, 12 h on – see Fig. S9) rather than continuous exposure, meaning comparable results would be obtained in a day-night cycling of a solar cell. To explore whether photothermal degradation was additive, we exposed a FA_0.83_Cs_0.17_Pb(I_0.7_Br_0.3_)_3_ film first to 85 °C for 12 hours and then subsequently to 470 nm illumination for 12 hours; the XRD intensity did not significantly decrease during this measurement (Fig. S10). Full data sets for all measurements carried out, including analysis of both peak widths and skews, can be found in SI, Note S3. In total we recorded the degradation of 22 thin-film perovskite samples from multiple batches to arrive at our conclusions. In summary, the degradation of lead-halide perovskite films is significantly faster under simultaneous exposure to heat and light than for either stressor alone, with comparable degradation mechanisms observed for different temperatures or excitation wavelengths (though we note UV rays can introduce additional degradation processes in films and devices).^62^ See Note S3 for further data and discussion of these points. Future stability testing of perovskite solar cells, for which intrinsic degradation of the absorber layer will always be a key factor, should therefore mainly focus on ISOS-L3 and LC3 tests rather than those based on only light or heat (for example, IEC damp heat tests).^63–66^

We now explore the mechanisms causing degradation under combined light (1 sun) and heat (85 °C) in detail. For this purpose, we analyse changes during degradation in PL intensity, PL mode (*i.e.* peak) and mean wavelength, XRD mode and mean 2*θ*, and XRD intensity about the halide perovskite peak. We present these data for thin films of FA_0.83_Cs_0.17_Pb(I_0.7_Br_0.3_)_3_ and FA_0.83_Cs_0.17_PbI_3_ in Fig. 2a/f, b/g, c/h and d/i, respectively. For the specific times indicated by coloured arrows and markers in Fig. 2a/f, we further plot full PL spectra and XRD peaks in Fig. 3 to allow for more detailed examination of changes in their shape. As discussed in detail below (and with further information in Note S4), our analysis reveals that for FA_0.83_Cs_0.17_Pb(I_0.7_Br_0.3_)_3_ degradation proceeds through three stages: halide segregation; A-site cation redistribution and fragmentation of the structure; and finally complete structural breakdown. For FA_0.83_Cs_0.17_PbI_3_ the process is comparable but halide segregation is absent, as expected. We proceed by discussing each phase and their underpinning mechanisms.

**Fig. 3:**
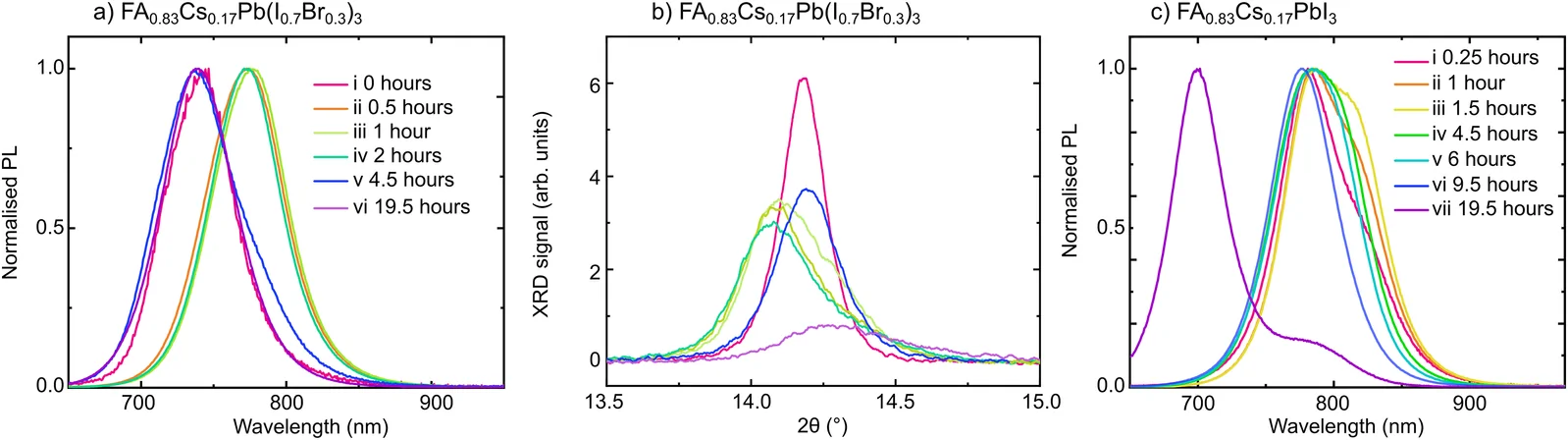
PL and XRD spectral evolution during perovskite degradation. (a) Normalised photoluminescence and (b) XRD signal for FA_0.83_Cs_0.17_Pb(I_0.7_Br_0.3_)_3_ when exposed to 85 °C and 470 nm at specific moments in the degradation cycle. Colours and roman numerals correspond to arrows in Fig. 2a. (c) Normalised photoluminescence for FA_0.83_Cs_0.17_PbI_3_ under the same conditions, with colours and roman numerals corresponding to arrows in Fig. 2f. Legend in (a) also applies to (b).

The initial phase of halide segregation in FA_0.83_Cs_0.17_Pb(I_0.7_Br_0.3_)_3_ films under photothermal stress is apparent in Fig. 2. Over the first ∼10 minutes, the PL intensity rapidly increases, the PL peak shifts to longer wavelengths, and a splitting in the mode and mean of the XRD peak becomes apparent with the XRD mean shifting to longer 2*θ* than the mode (implying that the XRD peak is no longer symmetric). Similar changes have previously been observed at room temperature^50,67^ and attributed to light-induced halide segregation,^47^ which leads to the formation of iodide- and bromide-rich domains. The resulting funnelling of charge carriers into iodide-rich regions enhances and red-shifts radiative recombination,^68,69^ as also observed here. Notably, we find that halide segregation in FA_0.83_Cs_0.17_Pb(I_0.7_Br_0.3_)_3_ films occurs significantly faster at 85 °C than at room temperature (as presented in Fig. S17) in contrast to what has been found previously for MAPb(I_1−*x*_Br_*x*_)_3_.^70^ No significant changes are observed for FA_0.83_Cs_0.17_PbI_3_ (Fig. 2f–i) over the same time window, also supporting our interpretation of the initial FA_0.83_Cs_0.17_Pb(I_0.7_Br_0.3_)_3_ dynamics relating to halide segregation.

Following the initial halide segregation, the next degradation phase of FA_0.83_Cs_0.17_Pb(I_0.7_Br_0.3_)_3_ films under photothermal stress is dominated by material strain and its interplay with A-cation distribution. While the XRD and PL peak positions now stabilize for approximately one hour (Fig. 2b and c) the peak width of the XRD signal slowly increases (Fig. S2), suggesting strain build-up or the formation of additional phases in the material.^71,72^ At ∼2 hours under photothermal stress, the PL peak shifts to much shorter wavelengths and becomes more asymmetric, *i.e.* the mode and mean are no longer equal (Fig. 2b). Conversely, for XRD the mode and mean return closer to their initial value and become approximately equal (and the strain also reduces, Fig. S2). This sudden shift can be seen directly by comparison of the dark green and blue lines in Fig. 3a and b, which indicate PL spectra and XRD peak patterns just before and after such changes have occurred (graphs for this entire transition window are shown in Fig. S18). We postulate that these effects are mainly caused by the diffusion and segregation of FA and Cs.^23^ Specifically, halide segregation will increase compositional inhomogeneities that induce local strain. When such strain becomes sufficiently large, FA and Cs may counteract this effect through diffusion. The resulting material exhibits a homogeneous lattice spacing (narrow symmetric XRD peaks) but heterogeneous composition of Cs–I and FA–Br rich regions (causing electronic heterogeneity apparent from broader and more asymmetric PL). The PL peak continues to shift to higher energy (smaller wavelength) after the change in XRD, which we attribute to reduced charge funnelling to lower bandgap regions. We suggest that this effect is caused by changes to the local electronic environment (potentially Cs- and FA-rich regions), or fracturing of the structure between Br- and I-rich domains that inhibits charge-carrier diffusion.^53^ The dynamic redistribution of FA and Cs thus becomes a second driver of bandgap variation across the structure, resulting in broader photoluminescence. These findings underline the importance of simultaneously monitoring XRD and PL during degradation tests, given that the observation of an unchanged XRD pattern clearly does not imply an unchanged material. Further discussion of this and other interpretations can be found in SI, Note S4.

For the single-halide FA_0.83_Cs_0.17_PbI_3_, the initial asymmetric PL shape (Fig. 2g and 3c for individual plots) suggests some initial compositional heterogeneity. The PL becomes more uniform at subsequent times, suggesting that diffusion of FA and Cs produces a more homogeneous structure. We propose that this process is comparable to annealing thin films which enables more uniform mixing of FA and Cs.^23^ Therefore, regardless of halide mixing, for both FACs perovskites, A-site cation diffusion is key during this phase of degradation. Diffusion of atoms to counteract strain has been observed in other thin-films solar absorbers with isovalent pairs, such as CIGS^73^ and is a main drawback of using multinary compounds.

At long exposure times (>8 hours), the degradation process becomes dominated by significant loss of crystalline material for both FA_0.83_Cs_0.17_Pb(I_0.7_Br_0.3_)_3_ and FA_0.83_Cs_0.17_PbI_3_. As shown in Fig. 2d and i, the XRD intensity of the perovskite peak begins to decline significantly, approaching near zero value around 20 hours. Importantly, for both materials decomposition varies in a highly nonlinear way. We find that outside of any initial halide segregation window, the degradation rate increases during the measurement to a maximum value at approximately mid-way through the degradation process, after which it decreases again, approaching 0 at long times (see Fig. S19a and b). This observation shows that models capturing perovskite device degradation^3–5,24,74^ need to move beyond assuming a linear absorber degradation process because, as we show here, the degradation rate is clearly not constant. We find that several sigmoidal models reproduce the change in XRD intensity well, with a full discussion presented in SI, Note S7 and fits for an autocatalytic model, where 
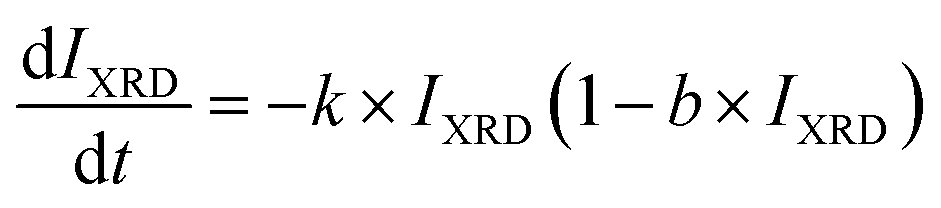
 (where *k* and *b* are fitting constants) presented in Fig. 2d and i. As is further discussed in SI, Note S7, degradation depends on both the fractions of degraded and pristine material. This finding potentially arises from degradation primarily occurring at the interface between these regions.

The last phase of decomposition is also accompanied by changes in PL wavelength and XRD angle. Between 8–20 hours of photothermal exposure, the PL peak (Fig. 2b) shifts to higher energy (smaller wavelength) for FA_0.83_Cs_0.17_Pb(I_0.7_Br_0.3_)_3_ and FA_0.83_Cs_0.17_PbI_3_, indicating increasing nominal bandgap. This finding is in agreement with the XRD peak (Fig. 2c) shifting to higher 2*θ* meaning formation of a structure with smaller lattice spacing. These observations suggest loss of one or both of iodine and FA from crystal structures,^75,76^ as is explored further below. Peculiarly, for FA_0.83_Cs_0.17_PbI_3_, at 16 hours we see a discontinuity in the PL mode (Fig. 2g) caused by the emergence of a shorter wavelength PL peak (Fig. 3c). We propose that this short wavelength PL originates from a caesium-rich phase. The rapid jump in PL, rather than a gradual shift, may suggest that a miscibility gap between FA and Cs-based halide perovskites opens up at this point.^77^

To investigate the end products of the complete perovskite decomposition process, we first examine changes in the full XRD pattern following 20 hours of photothermal degradation. We present initial and final (room temperature) XRD scans recorded immediately prior to and after such measurements in Fig. 2e and j. Here FA_0.83_Cs_0.17_Pb(I_0.7_Br_0.3_)_3_ initially corresponds to the cubic Pm3̄m space group (following assignments by Ruellou *et al.*),^43^ while FA_0.83_Cs_0.17_PbI_3_ has additional weak XRD peaks at 22.3° and 26.4° 2*θ*, likely related to the initial material being slightly inhomogeneous (as also evident in PL). For both halide perovskites, the final XRD scan demonstrates minimal remaining crystalline material. Surprisingly we see no evidence of PbI_2_ or metallic Pb structures, though these are likely present as small amorphous phases not detectable to XRD. We also observe no appearance of any other XRD peaks during stressing over this time scale.

We carried out *ex situ* optical microscopy of a pristine and a degraded FA_0.83_Cs_0.17_Pb(I_0.7_Br_0.3_)_3_ film, shown in Fig. 4a and b respectively, to further examine changes in film morphology following 20 hours of degradation. Two key points can be observed: firstly, the degraded material is significantly lighter in colour (compare Fig. 4b to a), suggestive of a wider bandgap or thinner material. Optical absorption measurements indeed demonstrate that the degraded film exhibits a larger bandgap and significantly more scattering, as shown in Fig. S20. Secondly, pinholes such as those shown in Fig. 4b have formed in the degraded film, while we observed no such regions in the pristine film shown in Fig. 4a (see Fig. S21 and S22 for lower magnification images of all sample types). Such pinholes suggest that material has left the perovskite film, potentially even breaking through the PMMA encapsulation layer. To understand film morphology at smaller length scales we used scanning electron microscopy (SEM). The pristine FA_0.83_Cs_0.17_Pb(I_0.7_Br_0.3_)_3_ film (Fig. 4c) shows a relatively standard polycrystalline morphology with sub-micrometer grain size. Conversely, the degraded material (Fig. 4d) was more challenging to measure in SEM due to local charging effects and exhibited no defined grain structure, with cracks and voids forming across its surface indicative of significant structural breakdown and presence of amorphous material. We present additional SEM images of the degraded sample in Fig. S23.

**Fig. 4:**
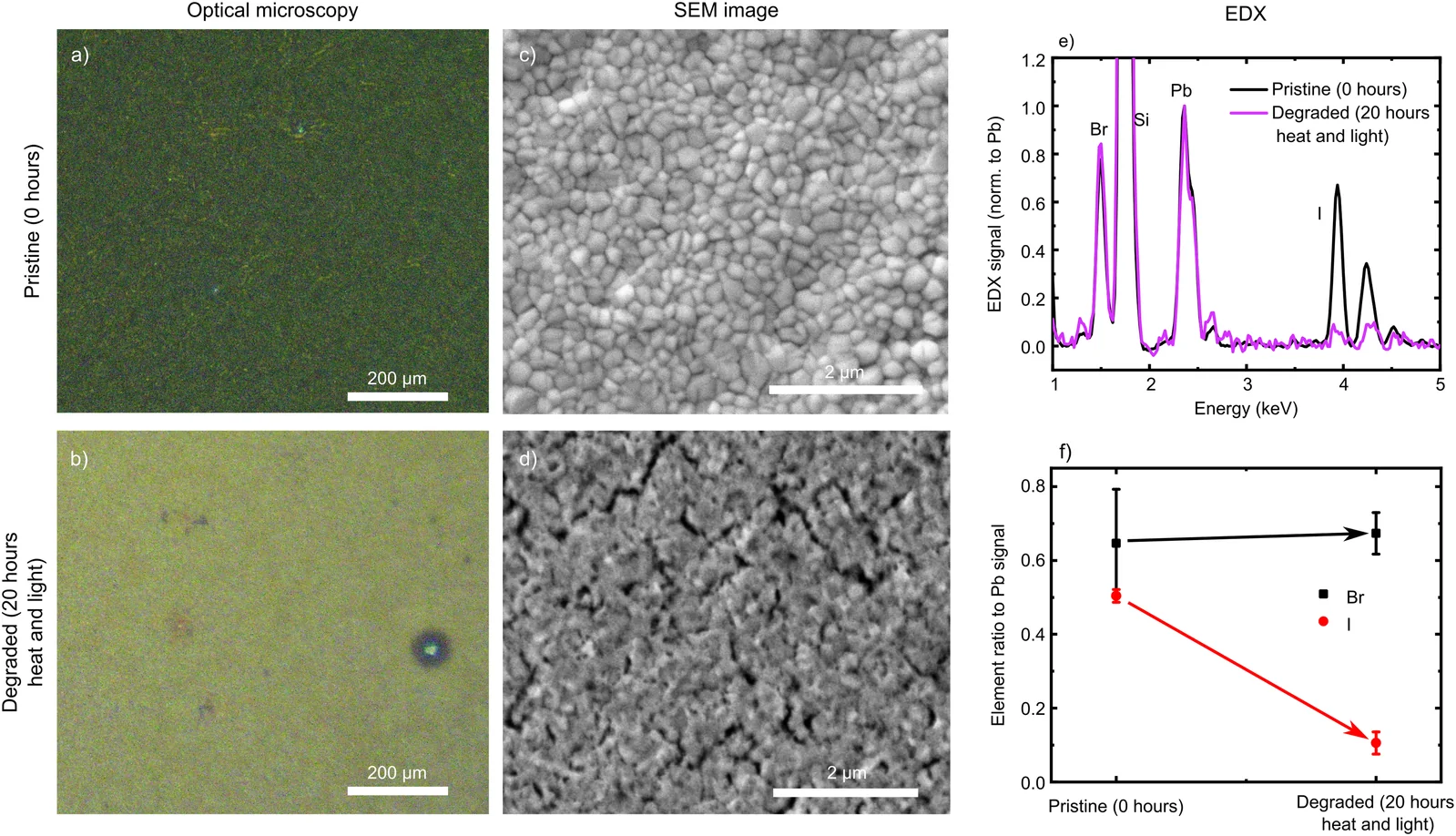
Optical, morphological and compositional degradation of perovskite films after 20 hours of heat and light. Optical/top-down electron microscopy images of (a)/(c) pristine and (b)/(d) degraded (20 hours at 85 °C under 470 nm illumination) FA_0.83_Cs_0.17_Pb(I_0.7_Br_0.3_)_3_ films. (e) Energy dispersive X-ray spectroscopy of cross sections of pristine and degraded FA_0.83_Cs_0.17_Pb(I_0.7_Br_0.3_)_3_, with signal strengths normalised to Pb. (f) Change in integrated peak ratio relative to Pb for bromine and iodine.

To examine any compositional changes occurring after 20 hours of photothermal exposure, we cracked a pristine and a degraded FA_0.83_Cs_0.17_Pb(I_0.7_Br_0.3_)_3_ sample in half to carry out cross-sectional energy dispersive X-ray (EDX) chemical mapping. Fig. 4e presents background-subtracted EDX signals normalised to the signal of the Pb, with all elements identified as labelled on the plot. While the bromine intensity is comparable for pristine and degraded films, the iodine signal decreases significantly relative to lead following degradation. Three measurements across different film locations on both pristine and degraded samples confirm this effect, as shown by the error bars in Fig. 4f. We note this finding is consistent with previous photovoltaic device degradation studies observing I_2_ outgassing under 1 sun and 80 °C.^78^ Overall, our results strongly support the picture emerging from Fig. 2 of structural collapse from loss of iodine, and potentially also FA,^75,76,79,80^ which EDX is unable to detect.

Overall, we find that the PL intensity varies remarkably non-monotonically over the 20 hours of photothermal exposure (Fig. 1c and 2a, f). Since PL intensity is often considered a key proxy for solar cell properties including open-circuit voltage,^81^ it would be insightful to understand the origins of such variations, which may include changes in morphology (and therefore photon out-coupling), charge-carrier funnelling and confinement in a fracturing landscape, thin-film absorption and charge trapping. We therefore carried out *in situ* time resolved photoluminescence (TRPL) measurements on FA_0.83_Cs_0.17_Pb(I_0.7_Br_0.3_)_3_, again while the sample was heated to 85 °C and illuminated by the same LED used in XRD measurements. To ensure comparability, we monitored the PL intensity during LED illumination and found similar changes to those shown in Fig. 2. We recorded TRPL by briefly turning off the LED and illuminating the sample with a pulsed laser (405 nm). We find that PL lifetimes follow a very similar trend to the PL intensity variation, with the overlay presented in Fig. S24. We note that variation in out-coupling would result in negligible variation in lifetimes and therefore cannot dominate such changes. Instead, our results suggest that changes in PL intensity during degradation are primarily caused by variation in trap-mediated recombination, which may also be influenced by absorption or increasing constraints on charge-carrier diffusion through the film.

Using the evidence presented above we now propose the dynamic sequence leading to FACs lead-halide perovskite photothermal degradation, which is shown schematically in Fig. 5a. At early times, halide segregation occurs in FA_0.83_Cs_0.17_Pb(I_0.7_Br_0.3_)_3_ resulting in the formation of iodine-rich and bromine-rich phases, which causes a rapid wavelength shift and intensity increase in PL. Continuing halide segregation causes strain to build up across the crystal structure, which is accompanied by a reduction in the PL intensity.^82^ At some point, strain is released through a compensating motion of A-site cations, giving more uniform lattice spacing and the formation of regions with enhanced PL (though in FA_0.83_Cs_0.17_Pb(I_0.7_Br_0.3_)_3_ we cannot rule out some concurrent motion of halides). As FA_0.83_Cs_0.17_Pb(I_0.7_Br_0.3_)_3_ continues to degrade, such regions become more electronically isolated (due to changes in the local electronic environment or fracturing of the material), preventing charge funnelling and resulting in multiple spectral contributions to the PL originating from different parts of the structure. Finally, gradual loss of iodine and potentially FA cause the formation of bromine-rich regions, associated with higher-energy PL and higher-angle XRD. Remaining perovskite regions at this point appear to be still cohesive and low in traps, giving reasonable PL intensity. However, further iodine and possibly A-site cation loss causes even these regions to degrade, triggering the final decrease in PL and XRD intensity. Overall, structural perovskite loss rates are first slow, then accelerate, and finally slow again in the late stages of degradation. Based on the sigmoidal form of XRD intensity change (Note S7) we propose that degradation is driven by interactions at interfaces between pristine and degraded regions. For FA_0.83_Cs_0.17_PbI_3_ we suggest that photothermal degradation follows a similar pattern, though no halide segregation can, of course, be observed. Therefore, in both FACs lead-halide perovskites degradation is driven by non-uniform mixing of A-site cations (and halides) and gradual loss of volatile compounds from the structure. Different film architectures may change the rate of this degradation – for example, the loss of volatile compounds may change with different encapsulation strategies.

**Fig. 5:**
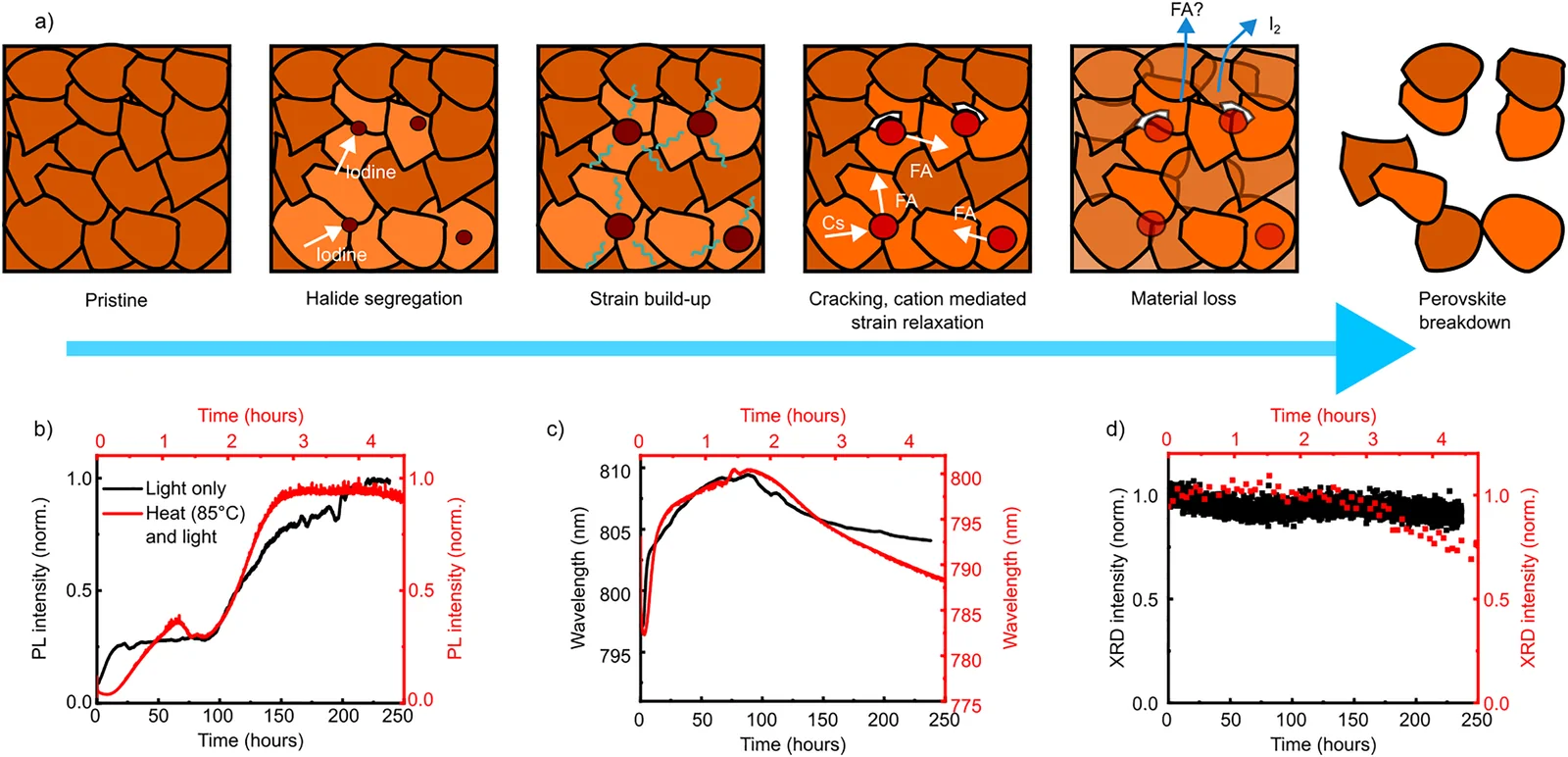
(a) Schematic of degradation dynamics observed for FA_0.83_Cs_0.17_Pb(I_0.7_Br_0.3_)_3_. For FA_0.83_Cs_0.17_PbI_3_ the second step (halide segregation) is not present but other processes proceed similarly. (b) Photoluminescence intensity, (c) photoluminescence wavelength and (d) XRD intensity normalised to initial 10 data points overlaid for two FA_0.83_Cs_0.17_PbI_3_ films, one exposed to 470 nm at room temperature (black lines and markers) and a second exposed to 85 °C and 470 nm (red lines and markers). Note that the two datasets in b–d are presented with respect to different time axes for room temperature (bottom, black) and 85 °C (top, red), with relative axes scaling (×53) determined from global fit to yield best overlap between the combined photoluminescence and XRD data sets. In addition, wavelength axes in sub-panel (c) are scaled separately because lattice expansion under heat affects the bandgap and therefore emission wavelength of the perovskite.

Finally, we discuss the use of elevated heat for accelerated lifetime testing of metal halide perovskites. Tests under 85 °C heat can serve two purposes: first they establish stability for operation in latitudes and locations where solar modules can routinely reach such temperatures under solar illumination.^83^ Second, they may allow extrapolation of anticipated device lifetimes under “normal” (*i.e.* lower ambient temperature) operation from data collected under elevated stress, such as heat. This latter practice of accelerated lifetime testing is a powerful way of determining readiness of materials for commercial deployment, which requires stability over decades. For example, for silicon, acceleration factors are typically on the order of 100–200 (and have been reported up to 5000) when moving from real-world locations to 85 °C and 85% humidity.^29,60,61,84^ Typically, acceleration factors are complex to extract for solar cells as different parts of modules can degrade at different rates. For emergent perovskite solar absorbers such acceleration factors have to date only been reported for specific device architectures, rather than individual materials,^4,24,33,74^ with Zhao *et al.* giving a summary of results.^3^ To understand material acceleration rates, we recorded *in situ* XRD and PL data continuously over 10 days for a FA_0.83_Cs_0.17_PbI_3_ film under 470 nm, 1 sun-equivalent illumination at ambient temperature (22 °C) and compared the obtained degradation dynamics with those recorded over one day for a fresh second sample under heat (85 °C) but otherwise identical conditions. We focus on this composition rather than FA_0.83_Cs_0.17_Pb(I_0.7_Br_0.3_)_3_ to avoid the additional complexities arising from halide segregation (see SI, Note S7). Fig. 5b–d presents such data recorded under heat in red, and those under ambient in black, with their respective time axes at the top and bottom scaled by a factor 53 obtained from a global fit to yield best overlap between the two XRD and PL data sets, as is discussed further in SI, Note S12. Both PL wavelength and intensity exhibit qualitatively similar dynamic responses (Fig. 5b and c) but elevating temperature to 85 °C accelerates degradation by approximately 53 times. This finding suggests that heat mainly accelerates degradation processes caused by light alone. Our value is comparable to those obtained in device stacks (ranging from 25–40),^3^ though we did not carry out an Arrhenius fit, because, as we have shown here, degradation is nonlinear. Nonetheless, our comparable values suggest that the absorber degradation processes we have explored here are an important cause of currently observed instabilities in full devices.^32,33^ XRD intensity similarly shows a scaled decline in intensity (Fig. 5d), with minimal change in the 2*θ* position over measurements (Fig. S26). The slight acceleration in XRD intensity decline at 85 °C on the scaled time axis at longer times, may potentially point to longer heat exposure opening additional degradation pathways. Overall, our findings, contrasted with existing literature on silicon solar cell degradation, suggest that application of elevated heat causes somewhat faster degradation in silicon than in metal halide perovskites.^29,60,61^ Application of light (*versus* dark) at a given temperature, on the other hand, is less detrimental to silicon than it is to these perovskites.^58,59^ Our findings present quantitative comparators for accelerated lifetime testing of metal halide perovskite absorber layers and complement device-scale studies to establish a predictive framework on accelerated lifetime testing of perovskite solar cell modules.

## Conclusion

We have revealed the fundamental degradation dynamics and mechanisms of device-relevant halide perovskites FA_0.83_Cs_0.17_Pb(I_0.7_Br_0.3_)_3_ and FA_0.83_Cs_0.17_PbI_3_ by recording *in situ* X-ray diffraction and photoluminescence over extended time periods. We demonstrate that under the combined stress of 1 sun illumination and elevated temperature (85 °C), degradation is substantially accelerated compared to the presence of either stressor alone. Degradation under such conditions progresses through three phases: halide segregation (for the mixed-halide perovskite), A-site cation diffusion in response to internal strain, and finally, loss of iodine and potentially FA at long times, leading to the disappearance of the crystalline perovskite structure. We showed that degradation dynamics are highly nonlinear, exhibiting an early slow phase that accelerates significantly in the interim degradation regime, while slowing again during terminal decline. This finding suggests that the currently common practice of linear extrapolation of early-time degradation data to extract lifetimes for perovskite materials or solar cells may lack predictive strength of actual long-term trends. Finally, degradation dynamics recorded over 10 days for solar illumination at ambient temperature compared with illumination at 85 °C suggest that this increased temperature accelerates degradation rates by a factor of 53. By revealing absorber-level degradation our work enables suitable design strategies for future material and device passivation. Our results underline the need for photothermal degradation testing on solar cells, reveal the nonlinear degradation pathways of halide perovskites, and enable a predictive framework to quantify accelerated lifetime testing for perovskite solar cells.

## Methods

### Halide perovskite film fabrication

#### FA_0.83_Cs_0.17_Pb(I_0.7_Br_0.3_)_3_

FAI (Greatcell Solar), CsI (Alfa Aesar) PbBr_2_ (Alfa Aesar), and PbI_2_ (TCI Chemicals) were combined in the desired molar ratio and dissolved in a 4 : 1 v/v ratio of DMF : DMSO (Sigma-Aldrich) to give a 1.3 M FA_0.83_Cs_0.17_Pb(I_0.7_Br_0.3_)_3_ perovskite ink. The ink was then spin coated onto z-cut quartz disks in a nitrogen-filled glovebox using a two-step deposition program: 1000 rpm for 10 s, followed by 6000 rpm for 35 s. 150 µL of anisole was dropped onto the substrate 35 s after the beginning of the spin coating. The films were then directly transferred to a hotplate and annealed at 100 °C for 60 min, after which they were allowed to cool to room temperature naturally. When the substrates were cool, a 15 wt% solution of polymethyl methacrylate (PMMA, Sigma-Aldrich, *M*_w_ ≈ 15 000) in chlorobenzene (Sigma-Aldrich) was spin coated onto the perovskite-coated substrate at 6000 rpm for 45 s. The samples were then kept in a nitrogen-filled glovebox until they were measured.

#### FA_0.83_Cs_0.17_PbI_3_

FAI (Greatcell Solar), CsI (Alfa Aesar) and PbI_2_ (TCI Chemicals) were combined in the desired molar ratio and dissolved in a 7 : 3 v/v ratio of DMF : DMSO (Sigma-Aldrich) to give a 1.3 M FA_0.83_Cs_0.17_PbI_3_ perovskite ink. The ink was then spin coated onto z-cut quartz disks in a nitrogen-filled glovebox using a three-step deposition program: 1000 rpm for 3 s, 3500 rpm for 10 s and 5000 rpm for 30 s. 150 µL of anisole was dropped onto the substrate 35 s after the beginning of the spin coating. The films were then directly transferred to a hotplate and annealed at 130 °C for 15 min, after which they were allowed to cool to room temperature naturally. When the substrates were cool a 15 wt% solution of polymethyl methacrylate (PMMA, Sigma-Aldrich, *M*_w_ ≈ 15 000) in chlorobenzene (Sigma-Aldrich) was spin coated onto the perovskite-coated substrate at 6000 rpm for 45 s. Using a surface profilometer, the thicknesses of the perovskite and the PMMA layers deposited onto glass were measured at ∼550 nm and 100 nm, respectively. Samples were kept in a nitrogen-filled glovebox until they were measured.

### X-ray diffraction

All X-ray measurements were carried out using a Rigaku SmartLab X-ray diffractometer. The incident X-ray radiation was the Cu-Kα lines and a HyPix-3000 camera recorded signal across a range of 2*θ* angles simultaneously. To increase signal strength no monochromator was used. Incident optics were chosen to ensure a beam size significantly smaller than the sample area, with total measured beam size being approximately 2 mm (direction perpendicular to 2*θ* scan) by 0.6 mm (direction partially in the plane of 2*θ* scans).

To process the *in situ* XRD scans, first an angular region where no signal was detected was selected (10.5° to 12°). For each scan the total counts in this region were found, and then the data were divided by this value – this approach corrects the data for any change in the incident XRD beam intensity over the measurement (typically minimal). Next, a second order polynomial was fit to the low- and high-angle edges of the first scan, using 10.3° to 12.5° and 15.5° to 16.0° data only (*i.e.* regions with no signal). This background was subtracted from all measurements. Data was then processed as discussed in the main text, with halide perovskite XRD intensity being the XRD counts summed from 12.5° to 15.5° (which only included the (100) halide perovskite signal). We note that due to the multinary (alloyed) nature of the halide perovskites studied, and clear existence of several compositional phases emerging during the degradation process, we did not find it suitable to carry out standard fits to XRD data (*e.g.* Rietveld refinement). Finally, the unchanged value of XRD peak intensity with time for samples exposed to only light or heat, as is presented in Fig. 1c and d, shows that XRD beam damage is not a significant concern in these measurements.

### Sample heating

For all XRD measurements the heating stage used was an Anton Paar DCS350 with a TCM100 temperature control unit, all built into the Rigaku diffractometer. For TRPL measurements a Linkam LTS420E PB6 M3 heating stage was used, coupled to a Linkam TCM100 temperature control unit.

For both heating stages, to ensure the sample was subject to the correct temperature, we first used a thermocouple (Fluke 80TK K-type) and thermal paste to measure the temperature of the hot-plate surface and compare it to the readout of the hot-plate's in-built thermocouple. We found these were in agreement. We then placed a bare quartz substrate on the heating stage and placed our thermocouple on the quartz surface (again using thermal paste). We varied the set-temperature of the hot-plate until we achieved either 85 ± 1 °C or 65 ± 1 °C on the quartz surface. Ambient room temperature was recorded as 22 ± 1 °C in all measurements. We anticipate that the temperature across the halide perovskite film is uniform and equal to that on the surface of the quartz substrate, as discussed in SI, Note S2.^85–87^

### Thermal camera and humidity

An FLIR C5 (version 1.2) was used in all measurements with software FLIR Thermal Studio Pro. We also recorded humidity in the XRD chamber prior to measurements, which was always between 35% and 45%.

### Sample illumination and photoluminescence detection during X-ray measurements

A custom-built attachment was connected to the Anton Paar heating stage, which mounted illumination, photoluminescence detection and the thermal camera. Samples were illuminated by continuous wave LEDs (Thorlabs M470L5 and M590L4 for 470 nm and 590 nm illumination respectively), with LEDs mounted so the incident wavefront was parallel to the sample plane. Light from the LEDs was collimated and focused onto the sample with achromatic lenses (Thorlabs AC254-100-AB-ML and AC254-30-AB-ML) and was also passed through a 650 nm shortpass and 450 nm longpass filter (Thorlabs FESH0650 and FELH0450 respectively) to ensure no bleed-through of other wavelengths. Lenses were adjusted to produce a spot size at the sample position comparable to the sample size (13 mm diameter quartz disks) and much larger than the XRD spot. Spot size was measured by a BFS-PGE-50S5M-C PoE GigE Blackfly® S Monochrome Camera mounted on an x–y–z stage, allowing several images to be taken and the entire beam shape to be stitched together. A typical spot shape, and discussion of the calculation of 1 sun illumination, is presented in SI, Note S13. Photoluminescence was recorded by collimation into an optic fiber which was connected to a spectrometer (Ocean Optics QEPro). Samples were illuminated immediately after reaching temperature in all cases.

### Time resolved photoluminescence

TRPL was recorded using a time correlated single photon counting setup. The PL wavelength of 750 nm was selected by a grating spectrometer (Princeton Instruments SP-2558) and detected using an MPD PDM single-photon avalanche detector (we note we saw similar results at other wavelengths). Events were binned using a PicoHarp 300 event timer. Samples were excited with a 405 nm Picoquant pulsed laser diode with repetition rate of 2.5 MHz or 1 MHz (rate chosen in each measurement to obtain best signal-to-noise ratio), with energy per pulse 15 pJ and major and minor ellipse diameters (for 
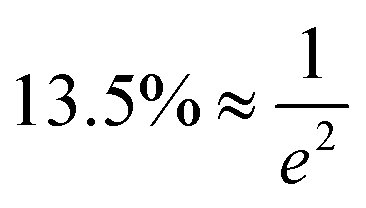
 clip level) of 1.8 mm and 2.0 mm (as recorded by a Thorlabs Dual Scanning Slit Beam Profiler BP209-VIS/M). For CW intensities counts were recorded on the same system, but this time using the same LED illumination as in XRD measurements.

### Optical microscopy

Optical microscopy was carried out using a Leica IVESTA I3. Exposure time and white balance were automatically set in all measurements.

### Scanning electron microscopy

Scanning electron microscopy (SEM) images were taken from a FEI Quanta 600 FEG instrument. Prior to all measurements, the chamber was pumped down to high vacuum with a pressure less than 2 × 10^−4^ mbar. For top-down SEM images an acceleration voltage of 5 kV was chosen, while for cross-sectional SEM images the acceleration voltage was set to 10 kV. Samples were washed in a chlorobenzene bath to remove the PMMA encapsulant prior to SEM images being recorded.

### Energy dispersive X-ray spectroscopy

We performed SEM energy dispersive X-ray spectroscopy (EDX) on the same FEI Quanta 600 FEG instrument system, using an Oxford Instruments detector. EDX profiles were scanned across cross-sections of studied thin films. To extract elemental information from EDX, we took repeated scans over areas of order of 100 µm^2^, each over a 100 s duration, using a 20 keV electron beam. The INCA software was used for data acquisition and analysis. We analysed EDX elemental spectra using the following characteristic X-ray lines corresponding to each element:^88^

Br Lα 1.481 keV

Si Kα 1.740 keV

Pb Mα 2.342 keV

I Lα 3.938 keV.

We used an asymmetric least-squares smoothing baseline fit to all EDX data to subtract background, producing the data presented in Fig. 4e (raw data is shown in Fig. S28). We then obtained the total intensity about each X-ray line and divided by the value for Pb to produce the data shown in Fig. 4f.

### Absorption measurements

Transmission and reflection were measured using a Fourier transform infrared (FTIR) Spectrometer (Bruker Vertex 80v), with a reference of nothing (air) used in transmission. The excitation source was a tungsten halogen lamp, the detector was silicon and a CaF_2_ beamsplitter was used in the excitation path.

## Conflicts of interest

There are no conflicts of interest.

## Supplementary Material

EE-019-D6EE04175B-s001

## Data Availability

The data underlying this manuscript are available from the corresponding author at reasonable request. Supplementary information (SI) is available and includes a note on PMMA encapsulation; a note on temperature across the perovskite during the measurement; additional XRD and PL results; a discussion on data interpretation; comparison of halide segregation rates at different temperatures; further discussion of fitting experimental XRD data; sample absorption measurements; optical images of pristine and degraded films; SEM images of degraded films; overlay of steady-state and time-resolved luminescence during degradation; discussion of the extraction of the acceleration factor; discussion of the LED illumination; and raw EDX data. See DOI: https://doi.org/10.1039/d6ee04175b.
