# Supplementary material for: Degradation dynamics of lead-halide perovskites under combined heat and light
Source: Energy Environ Sci. 2026 Sep 17;19(20):7158–70. doi: 10.1039/d6ee04175b (PMC13632668; doi:10.1039/d6ee04175b)
Supplement: Supplementary file 1 [file EE-019-D6EE04175B-s001.pdf]

Supplementary Information (SI) for Energy & Environmental Science.
This journal is © The Royal Society of Chemistry

Supplementary Information: Degradation dynamics of lead halide perovskites under combined heat and light

Alan R. Bowman[1], Qimu Yuan[1], Joshua R. S. Lilly[1], Adam T. M. Wood[1], Vincent J.-Y. Lim[1], Michael B. Johnston[1], Nakita K. Noel[1] and Laura M. Herz[1+]

1. Department of Physics, University of Oxford, Clarendon Laboratory, Parks Road, Oxford, OX1 3PU, United Kingdom

+corresponding author: laura.herz@physics.ox.ac.uk

Supplementary Information Note 1 – use of PMMA as an encapsulation layer

We used a PMMA layer to encapsulate all films reported on in the main text. PMMA is highly resistant to moisture and a reasonable oxygen encapsulant as well[1]. This situation is comparable to that deployed in many device degradation studies reported in academia and industry, where the edge sealants deployed still allow for some oxygen ingress[2]. Because the XRD intensity does not change during our heat-only and light-only stressing tests (Figs. 1c, d) we can conclude that PMMA is working well as an encapsulation layer in our measurements. At long times in the degradation process it is possible that some breaks or pinholes form not only in the perovskite, but also in the encapsulation layer (c.f. Fig. 4). We note that similar effects will also occur in full devices, for which at the final degradation steps cracks may form that propagate through several device layers, potentially even through the encapsulation layer[3]. Therefore, both for the half-stacks explored here, and for studies reported on full devices, one cannot fully exclude the role of oxygen or moisture at long times. The acceleration factor we obtain in Fig. 5 is comparable to that reported in the literature for full devices[4,5], suggesting that our approach is valid. Finally, we note that PMMA is one of the few materials that is transparent to both X-rays and light, making it the ideal encapsulation layer for our system.

Supplementary Information Note 2 – thermal camera response

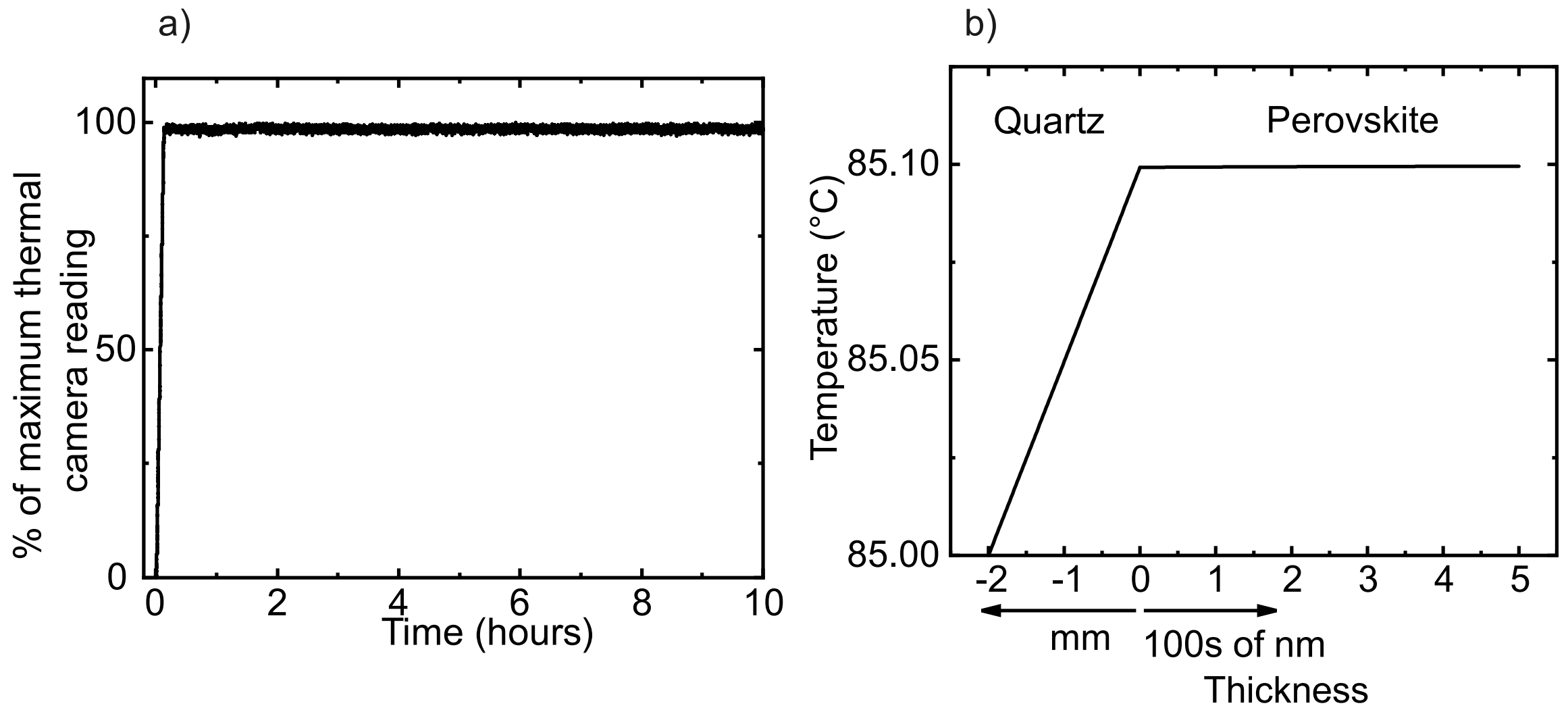

Fig. S1. a) Example of thermal camera output. Small oscillations in temperature are caused by periodic recalibrations of the thermal camera by the software. b) Simulation of temperature profile across the quartz and perovskite, when the quartz base is at 85°C and 1-sun illumination is incident on the sample.

Thermal cameras measure irradiance, which is typically converted to temperature by knowledge of the sample's emissivity in the mid-IR. However, as emissivity is unknown for our samples, in Fig. S1a we plot the converted temperature values as a percentage of the maximum temperature. This prevents us having to assume an emissivity value. The data confirm that temperature is not changing during the measurement. Additionally, in this measurement we turned on the light source ~ 5 minutes after the heating stage reached temperature, and we saw no change in the recorded signal i.e. light incident on the sample did not change the recorded temperature.

In Fig. S1b we plot the simulated temperature profile of a halide perovskite on quartz when the temperature of the quartz base is set to 85°C and 1-sun illumination is incident on the halide perovskite. Negligible temperature variation across the whole system is observed, in agreement with the thermal camera reading in Fig. S1a. In our simplified one-dimensional model the only heat flux permitted is through the base of the 2 mm thick quartz substrate. The heating caused by the incident beam of light is assumed uniform across the whole 500 nm thick perovskite layer, and thermal conductivities were set to 10.7 WmK^{-1} for quartz and 0.5 WmK^{-1} for the perovskite, based on literature values[6–8]. In summary, the high thermal conductivity of the quartz prevents significant temperature variation across the halide perovskite.

Supplementary Information Note 3 – other XRD degradation results

We plot the peak (mean and mode), intensity, peak width and Pearson skew of PL and XRD data for all measurements discussed in the main text. The peak width was calculated as the standard deviation of the data, representing the spread of the data about the mean. The Pearson skew is defined as $\frac{\text{Mean}-\text{Mode}}{\text{Standard deviation}}$. A value of 0 suggests an approximately symmetric peak shape, a positive value a skew to larger wavelengths/2θ values, and a negative value a skew to smaller wavelengths/2θ values. We use this metric because multiple phases are present in the materials during measurements, as discussed in the main text.

1. **$FA_{0.83}Cs_{0.17}Pb(I_{0.7}Br_{0.3})_3$ results**

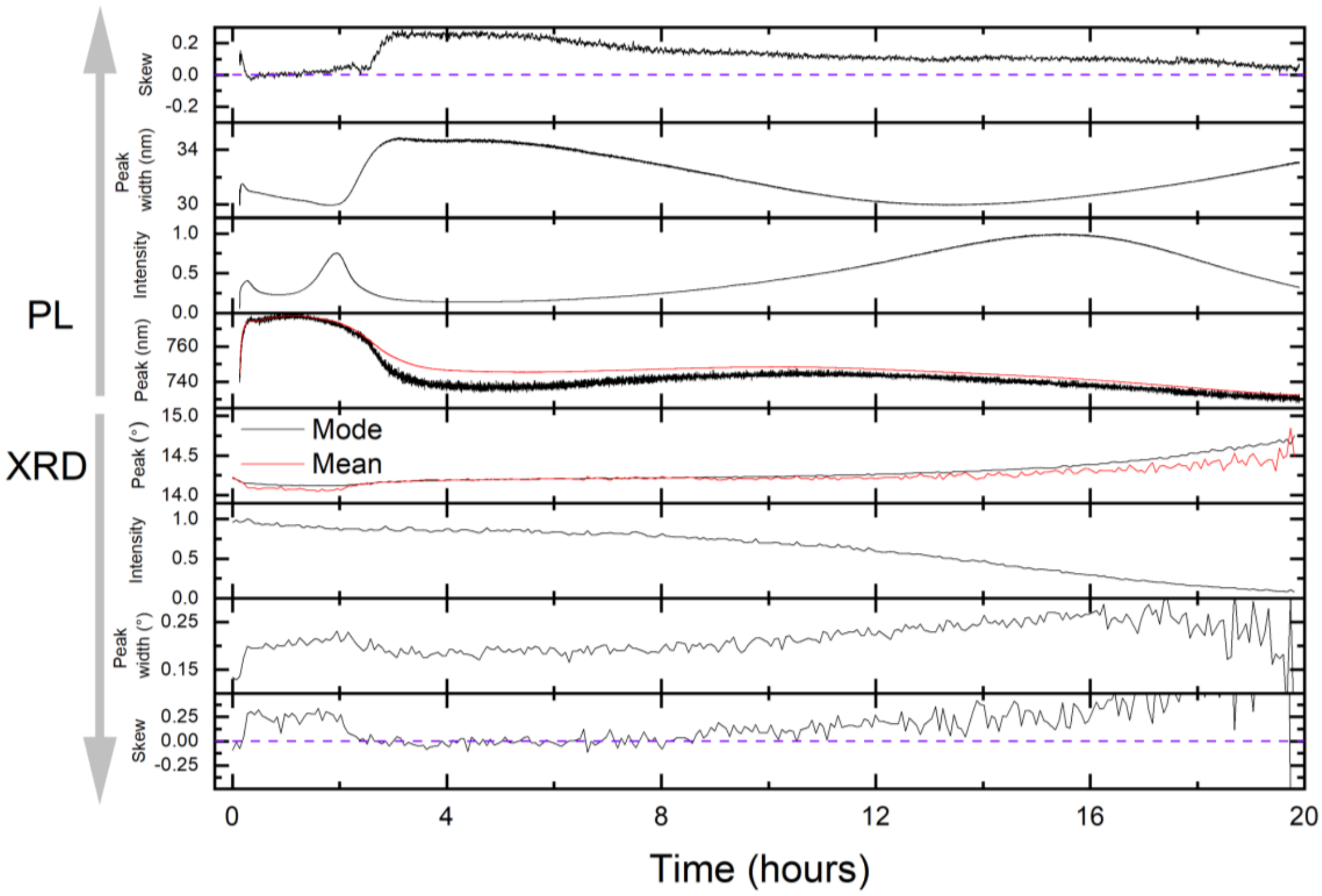

Fig. S2. Degradation dynamics during continuous exposure of $FA_{0.83}Cs_{0.17}Pb(I_{0.7}Br_{0.3})_3$ to 85°C and 470 nm, 1-sun equivalent illumination over 20 hours, showing (from the centre up/down): the peak (mean and mode), intensity, peak width and Pearson skew extracted from PL (top half) and XRD (bottom half) data.

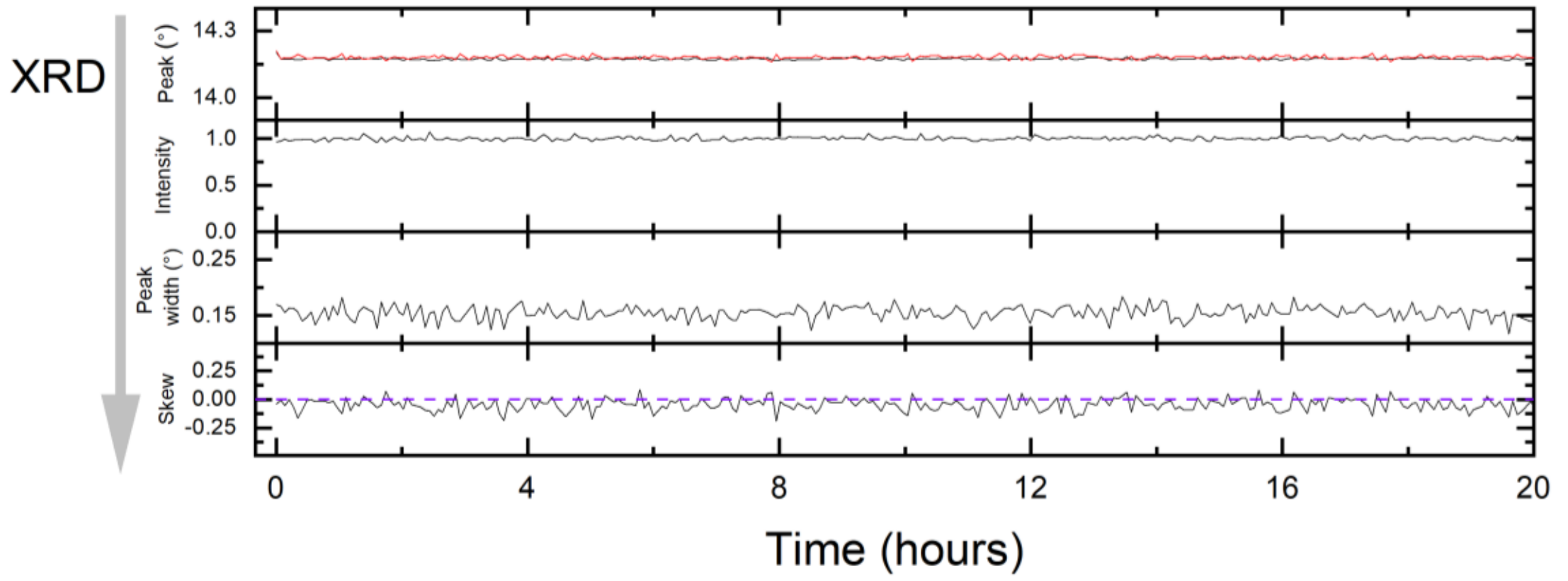

Fig. S3. Degradation dynamics during continuous exposure of $FA_{0.83}Cs_{0.17}Pb(I_{0.7}Br_{0.3})_3$ to 85°C over 20 hours, showing (from the top downwards): the peak (mean and mode), intensity, peak width and Pearson skew extracted from XRD data. No PL results are shown as the sample was not illuminated.

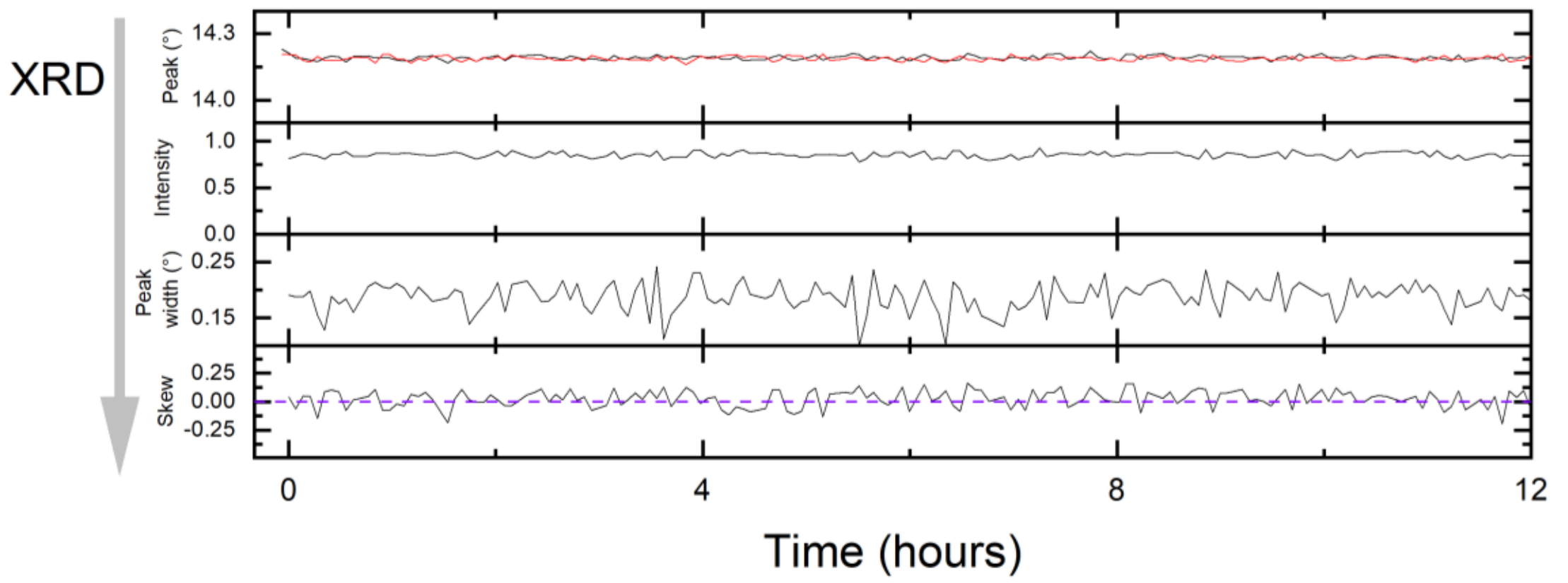

Fig. S4. A second measurement of degradation dynamics during continuous exposure of $FA_{0.83}Cs_{0.17}Pb(I_{0.7}Br_{0.3})_3$ to 85°C over 12 hours, showing (from the top downwards): the peak (mean and mode), intensity, peak width and Pearson skew extracted from XRD data. No PL results are shown as the sample was not illuminated.

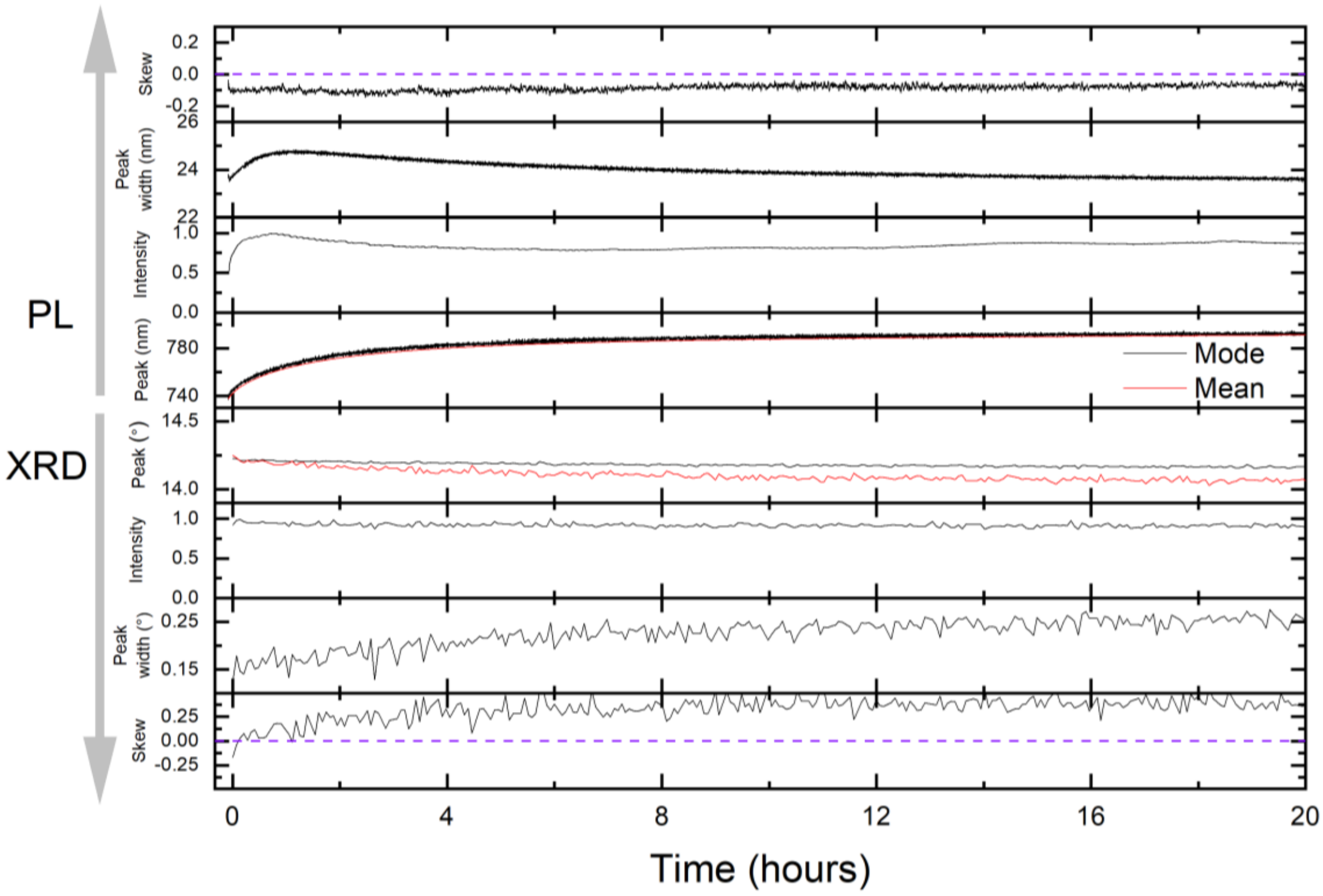

Fig. S5. Degradation dynamics during continuous exposure of $FA_{0.83}Cs_{0.17}Pb(I_{0.7}Br_{0.3})_3$ to 470 nm, 1-sun equivalent illumination over 20 hours at ambient temperature (22°C), showing (from the centre up/down): the peak (mean and mode), intensity, peak width and Pearson skew extracted from PL (top half) and XRD (bottom half) data. PL shifts to longer wavelength and XRD mean and mode split, as previously discussed by others[9]. We note this film was from a second batch so degradation processes have slightly different strengths.

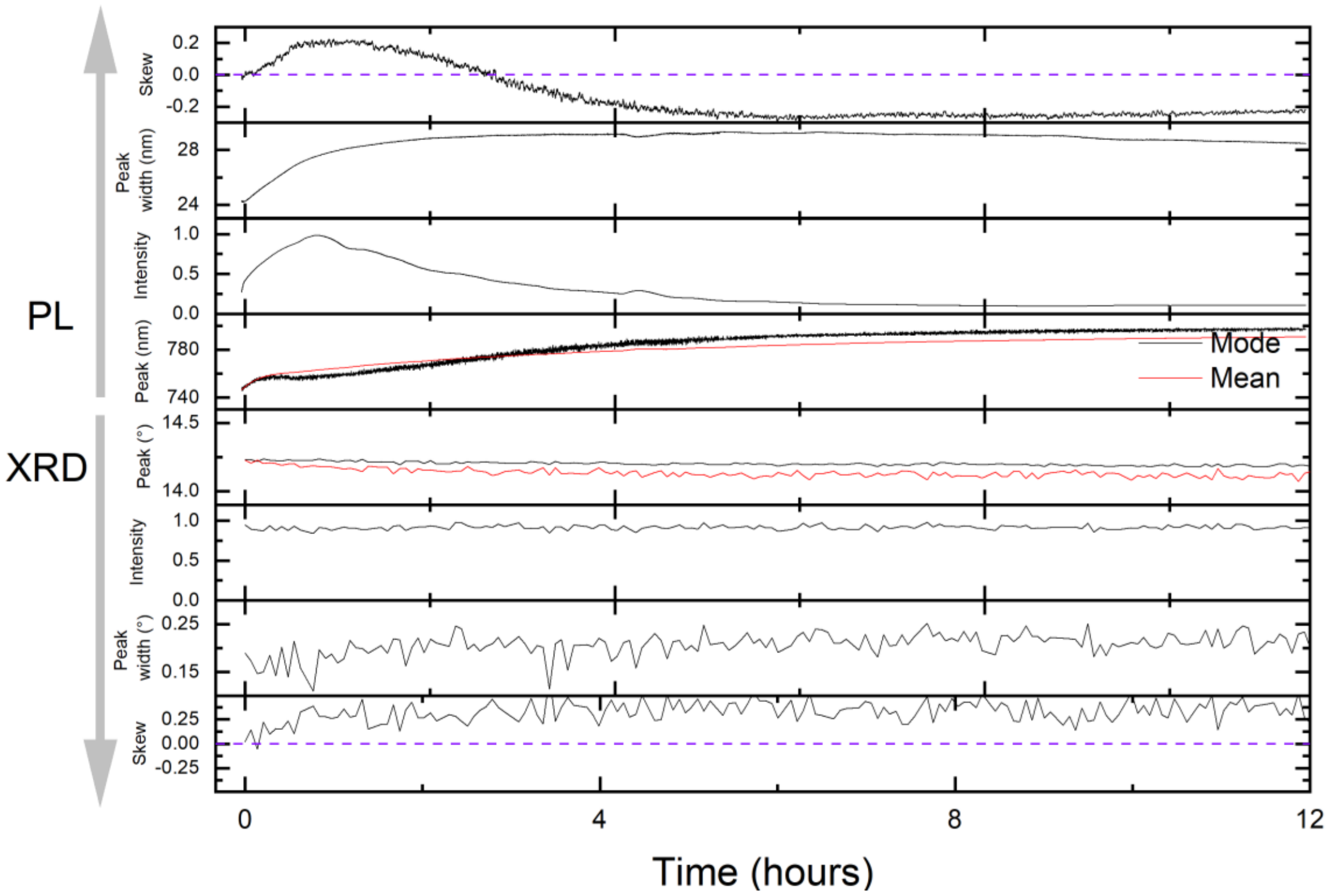

Fig. S6. A second example of degradation dynamics during continuous exposure of $FA_{0.83}Cs_{0.17}Pb(I_{0.7}Br_{0.3})_3$ to 470 nm, 1-sun equivalent illumination over 12 hours at ambient temperature (22°C), showing (from the centre up/down): the peak (mean and mode), intensity, peak width and Pearson skew extracted from PL (top half) and XRD (bottom half) data. PL shifts to longer wavelength and XRD mean and mode split, as previously discussed by others[9].

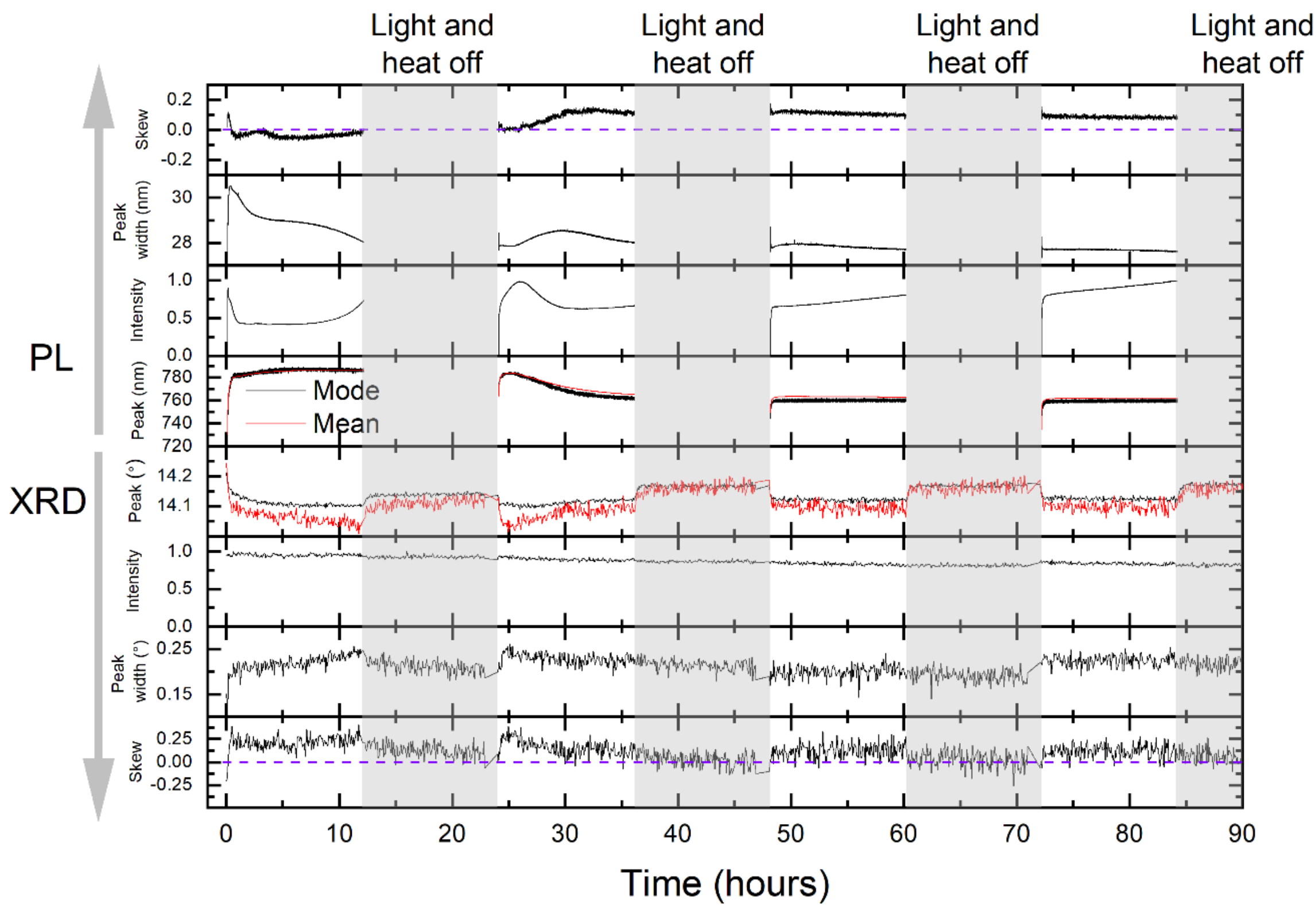

Fig. S7. Degradation dynamics during cycled (12 hours on, 12 hours off) exposure of $FA_{0.83}Cs_{0.17}Pb(I_{0.7}Br_{0.3})_3$ to 65°C and 470 nm, 1-sun equivalent illumination over 90 hours, showing (from the centre up/down): the peak (mean and mode), intensity, peak width and Pearson skew extracted from PL (top half) and XRD (bottom half) data.

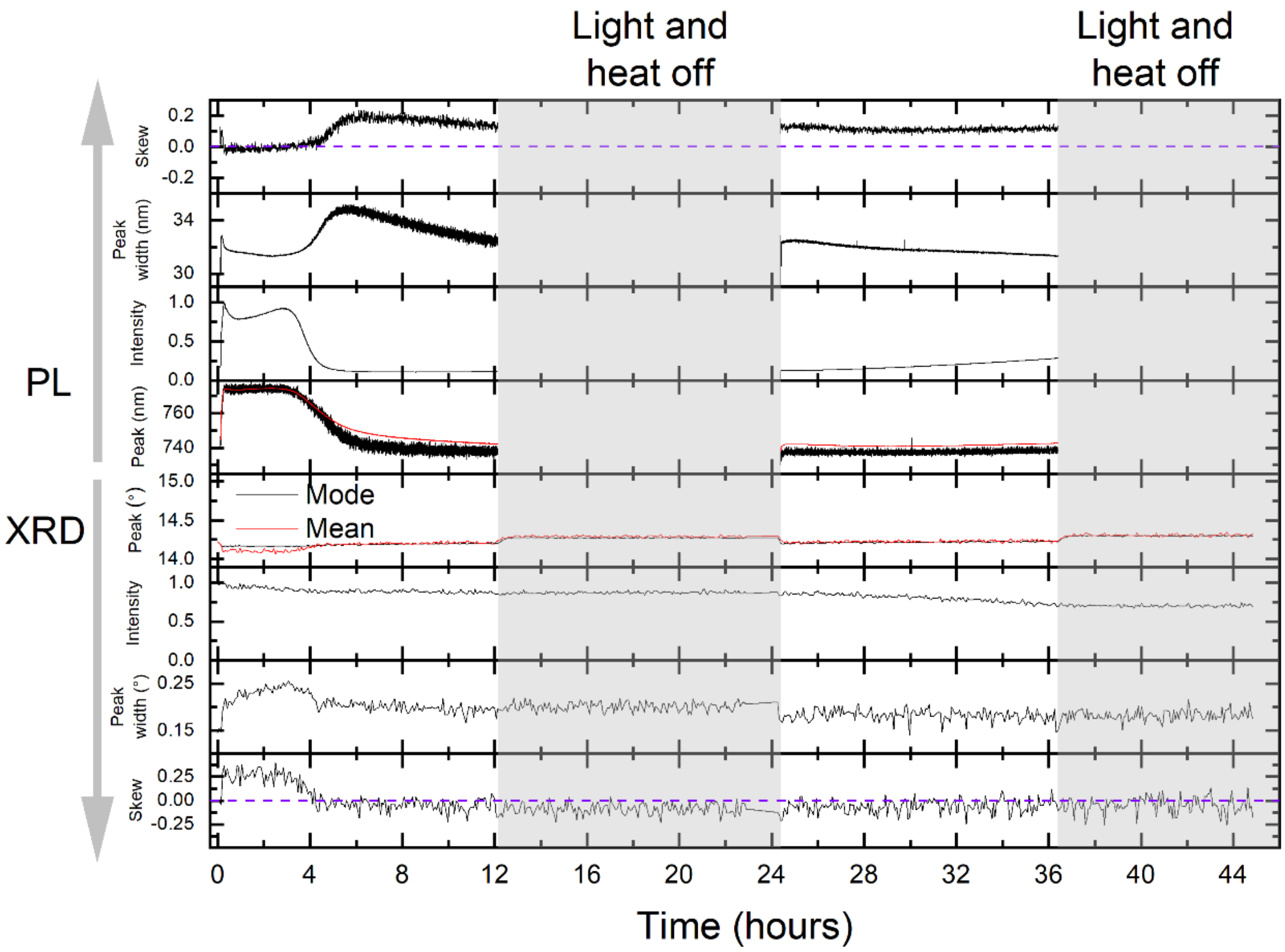

Fig. S8. Degradation dynamics during cycled (12 hours on, 12 hours off) exposure of $FA_{0.83}Cs_{0.17}Pb(I_{0.7}Br_{0.3})_3$ to 85°C and 590 nm, 1-sun equivalent illumination over 45 hours, showing (from the centre up/down): the peak (mean and mode), intensity, peak width and Pearson skew extracted from PL (top half) and XRD (bottom half) data. We do not anticipate longer-term degradation processes to be fully identical as while the intensity is set to ensure the same number of photons are absorbed in the initial halide perovskite, at subsequent times the strength of photon absorption at 590 nm may not be the same as for 470 nm illumination.

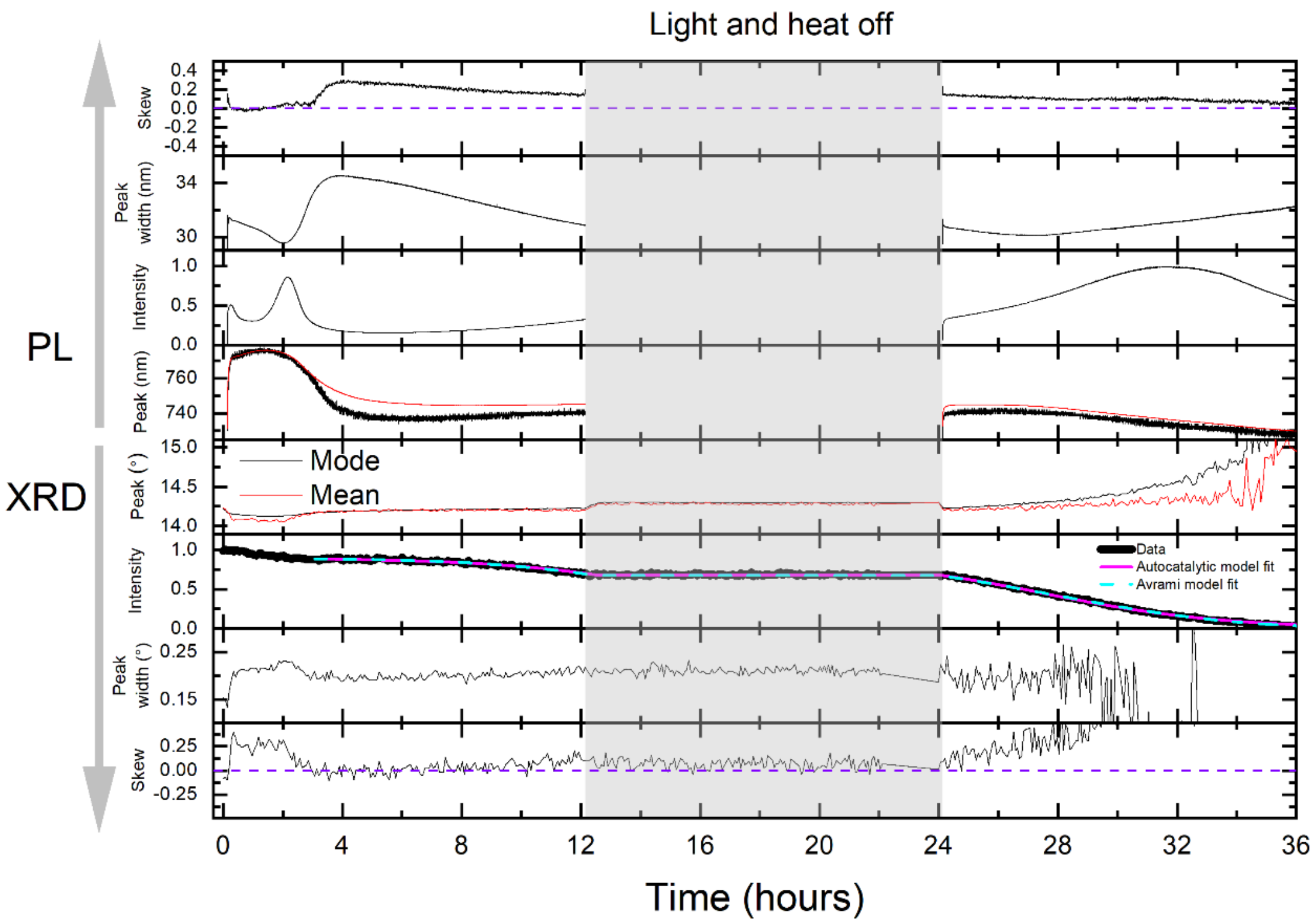

Fig. S9. Degradation dynamics during cycled (12 hours on, 12 hours off) exposure of $FA_{0.83}Cs_{0.17}Pb(I_{0.7}Br_{0.3})_3$ to 85°C and 470 nm, 1-sun equivalent illumination over 36 hours, showing (from the centre up/down): the peak (mean and mode), intensity, peak width and Pearson skew extracted from PL (top half) and XRD (bottom half) data. Results are comparable to those shown in Fig. S2 but with a 12 hour pause when heat and light exposure is stopped. Fits to XRD intensity data discussed in Supplementary Information Note 7.

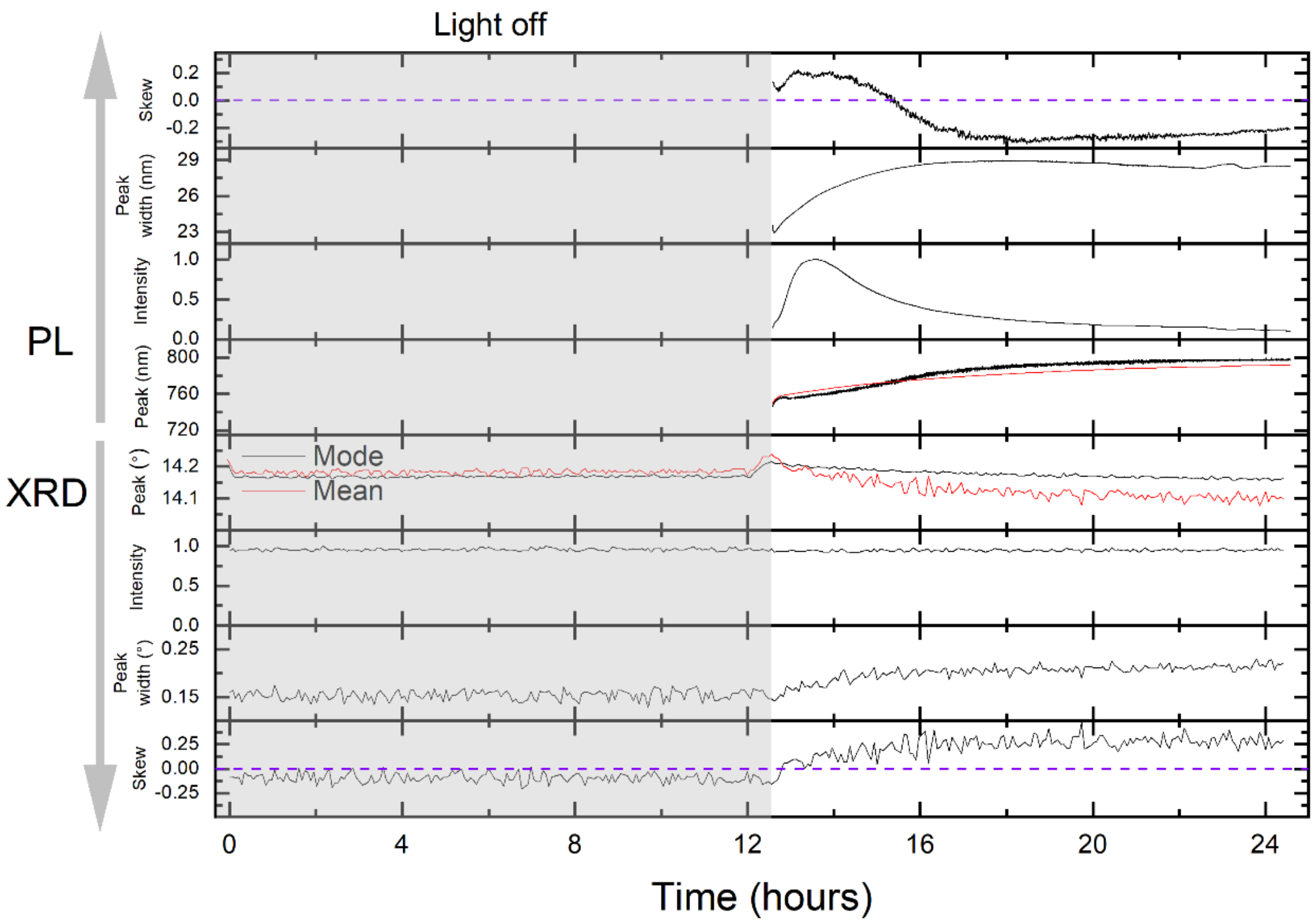

Fig. S10. Degradation dynamics during exposure of $FA_{0.83}Cs_{0.17}Pb(I_{0.7}Br_{0.3})_3$ to 85°C for 12 hours, and then 470 nm for 12 hours (at room temperature), showing (from the centre up/down): the peak (mean and mode), intensity, peak width and Pearson skew extracted from PL (top half) and XRD (bottom half) data.

2. $FA_{0.83}Cs_{0.17}PbI_3$

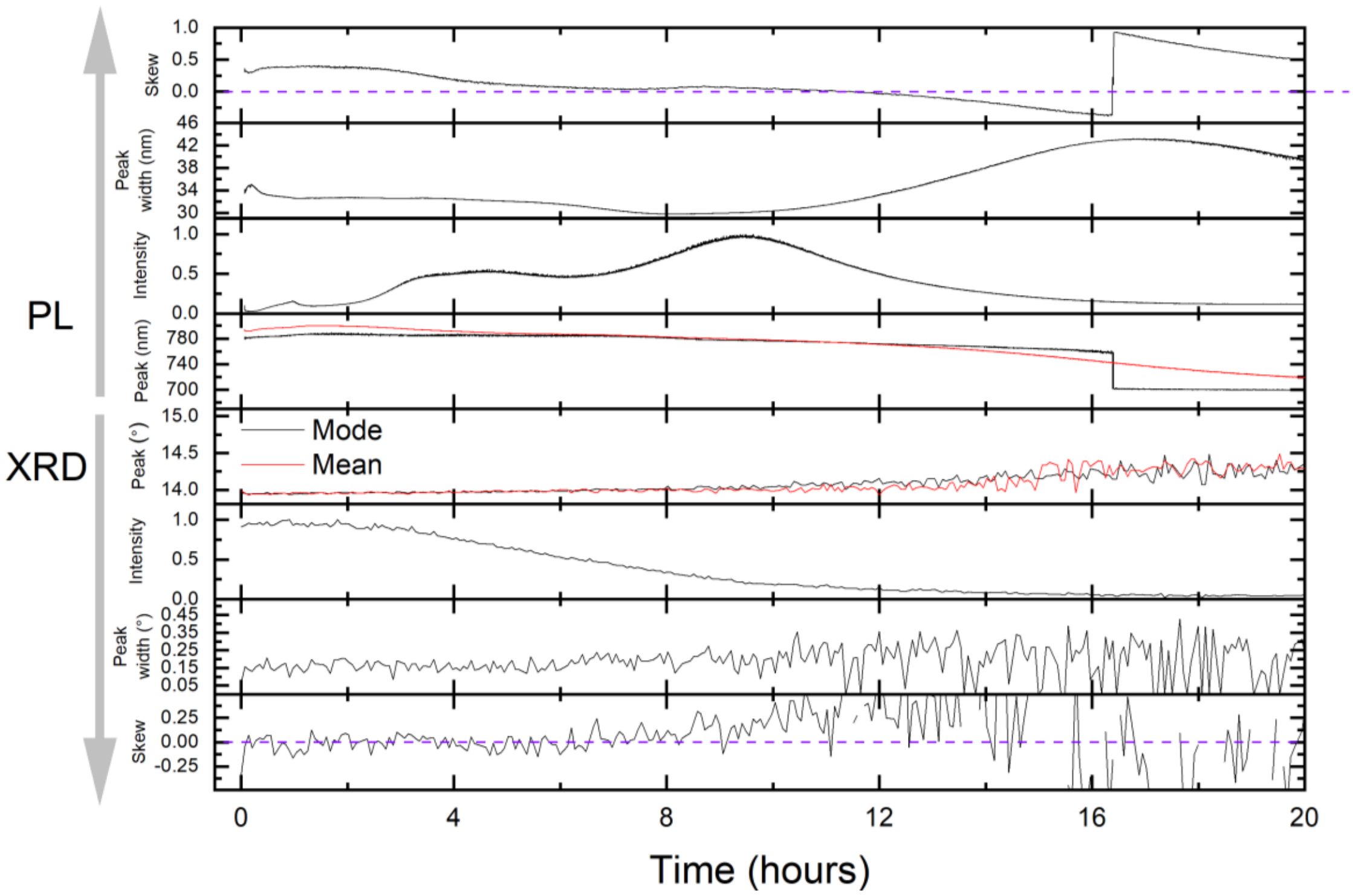

Fig. S11. Degradation dynamics during continuous exposure of $FA_{0.83}Cs_{0.17}PbI_3$ to 85°C and 470 nm, 1-sun equivalent illumination over 20 hours, showing (from the centre up/down): the peak (mean and mode), intensity, peak width and Pearson skew extracted from PL (top half) and XRD (bottom half) data.

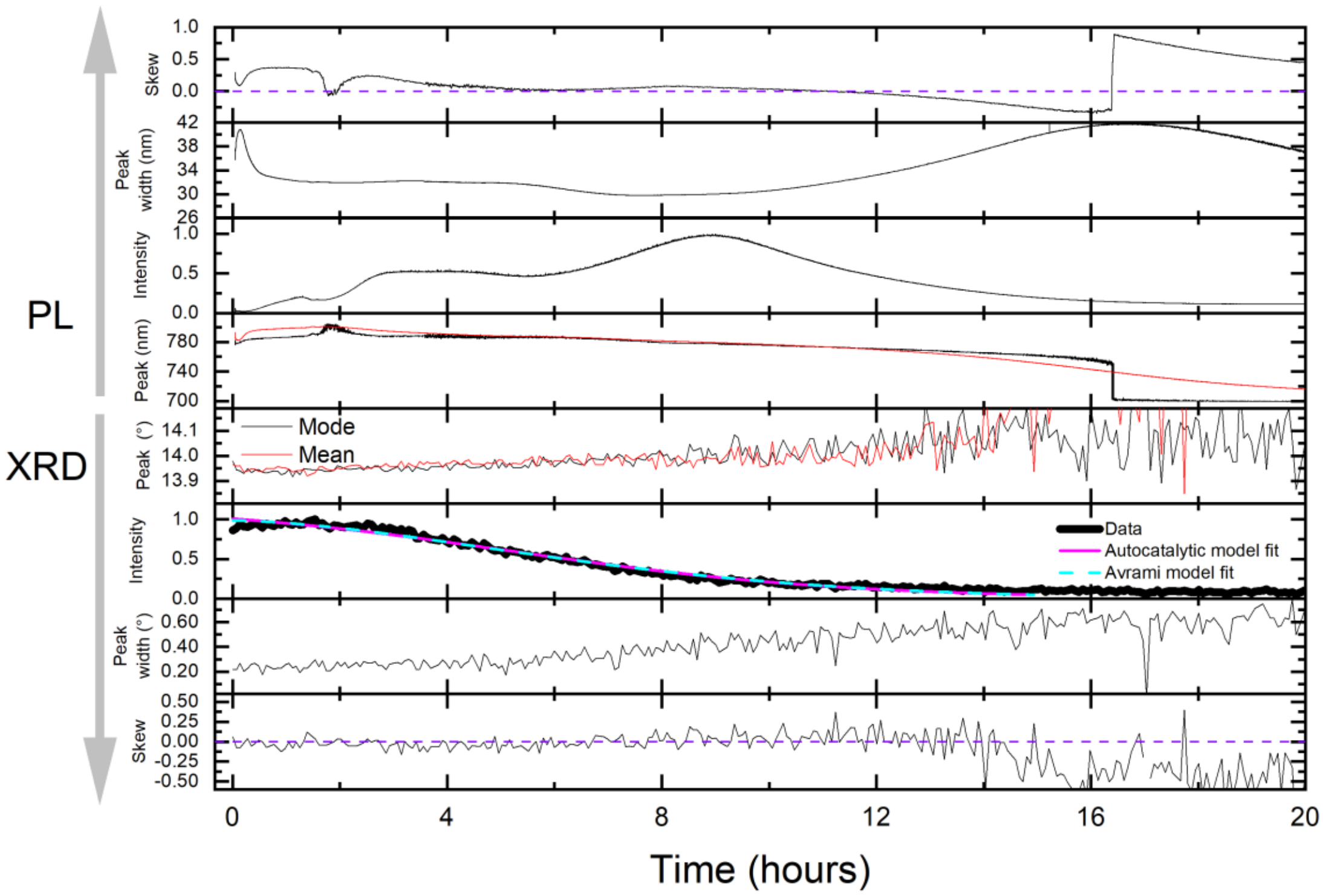

Fig. S12. Repeat measurement of degradation dynamics during continuous exposure of $FA_{0.83}Cs_{0.17}PbI_3$ to 85°C and 470 nm, 1-sun equivalent illumination over 20 hours, showing (from the centre up/down): the peak (mean and mode), intensity, standard deviation and Pearson skew extracted from PL (top half) and XRD (bottom half) data. Fits to XRD intensity data discussed in Supplementary Information Note 7.

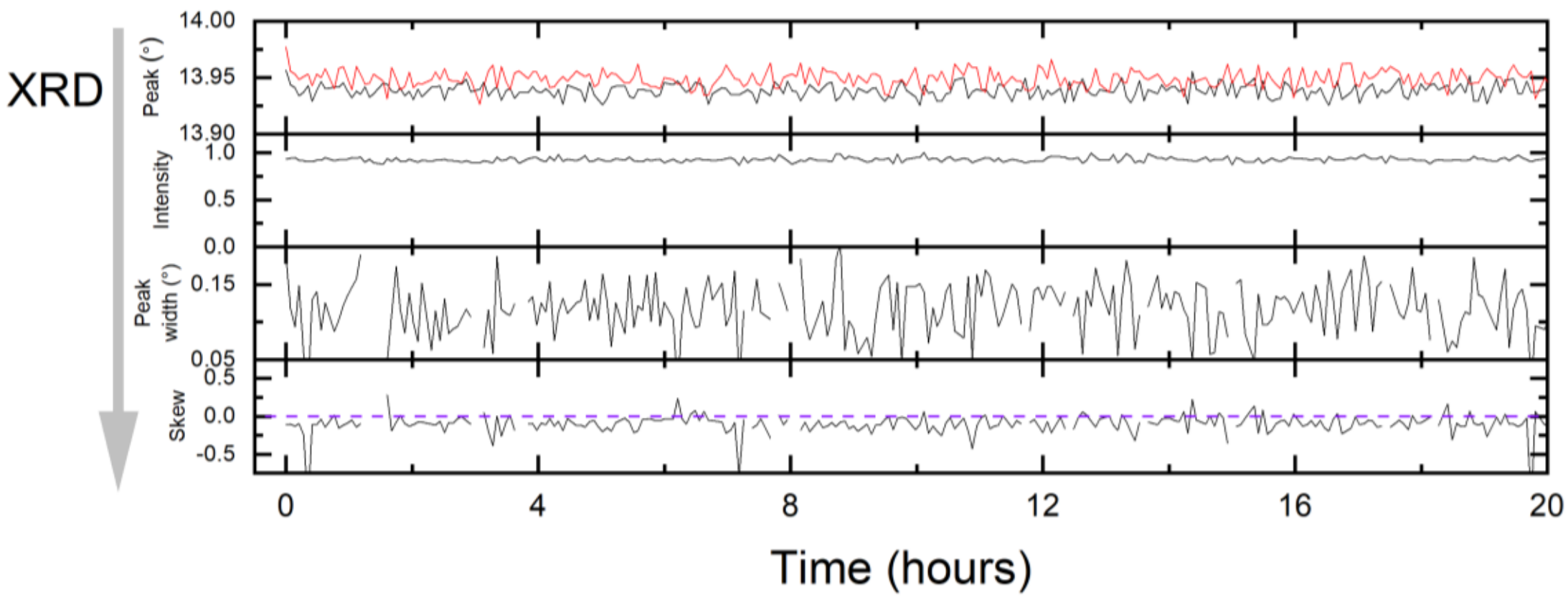

Fig. S13. Degradation dynamics during continuous exposure of $FA_{0.83}Cs_{0.17}PbI_3$ to 85°C over 20 hours, showing (from the top downwards): the peak (mean and mode), intensity, standard deviation and Pearson skew extracted from XRD data. No PL results are shown as the sample was not illuminated. Missing data points in peak width and skew are due to negative signal contributions far from the perovskite peak.

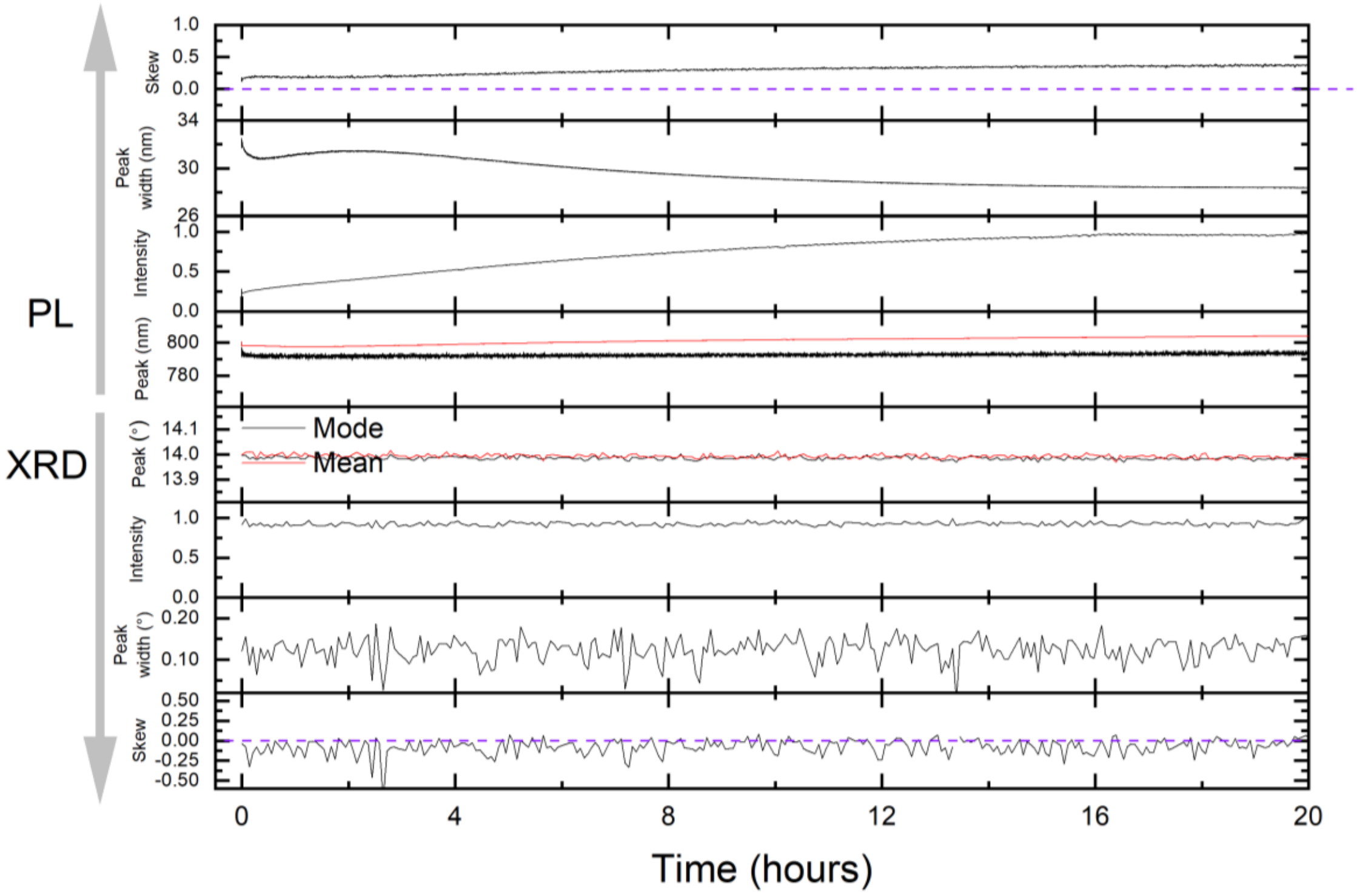

Fig. S14. Degradation dynamics during continuous exposure of $FA_{0.83}Cs_{0.17}PbI_3$ to 470 nm, 1-sun equivalent illumination over 20 hours at ambient temperature (22°C), showing (from the centre up/down): the peak (mean and mode), intensity, peak width and Pearson skew extracted from PL (top half) and XRD (bottom half) data.

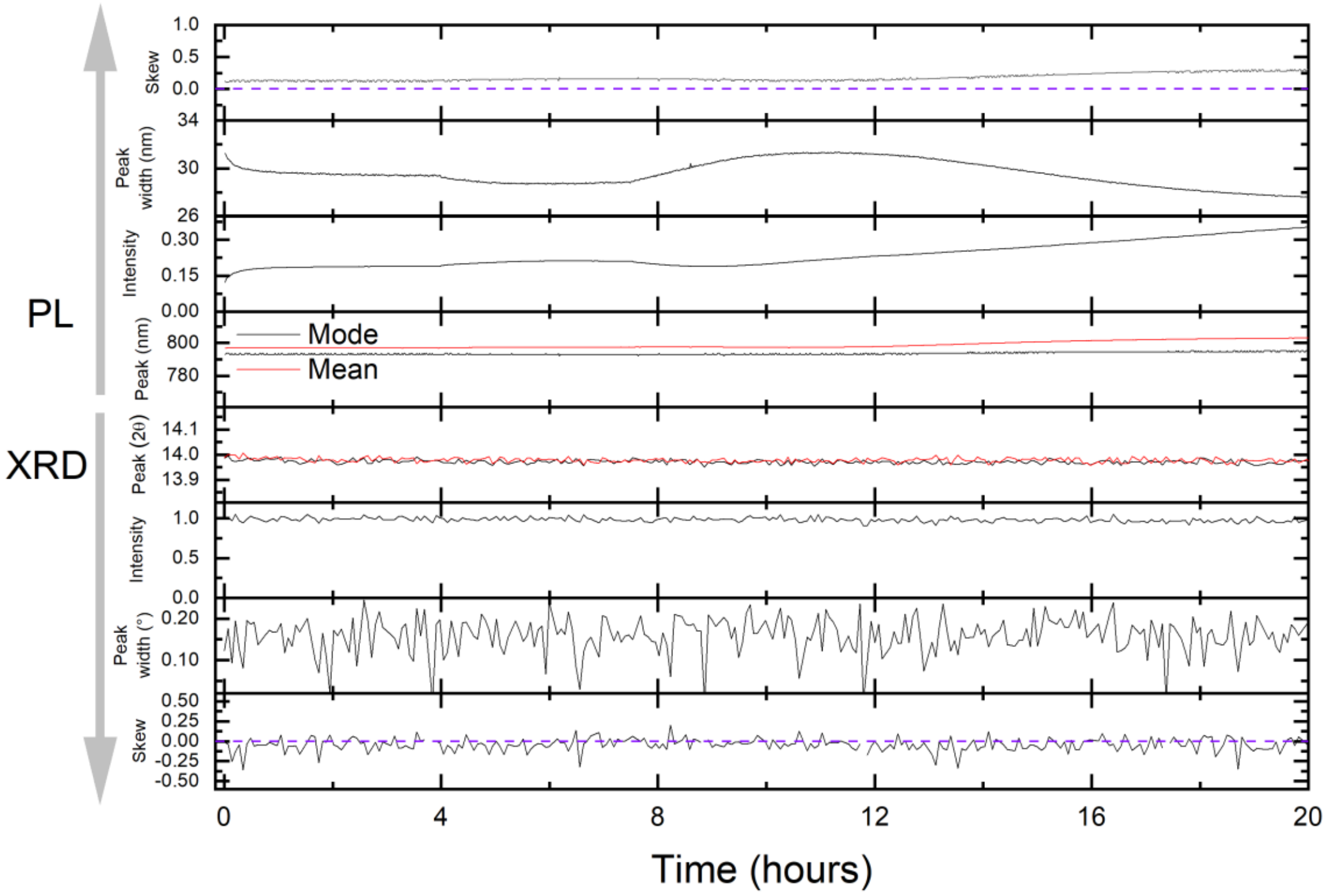

Fig. S15. Degradation dynamics during continuous exposure of $FA_{0.83}Cs_{0.17}PbI_3$ to 470 nm, 1-sun equivalent illumination showing the first 20 hours of a 240 hour scan at ambient temperature (22°C), showing (from the centre up/down): the peak (mean and mode), intensity, peak width and Pearson skew extracted from PL (top half) and XRD (bottom half) data.

We finally discuss in more detail whether excitation at different wavelengths makes a difference to degradation dynamics. The excitation density we calculated and set for each wavelength (Supplementary Information Note 13) relies on the absorption coefficient recorded for the pristine (undegraded) perovskite film. Therefore, we expect degradation rates to be identical at early times when light absorption mostly reflects that of the pristine structure, while at subsequent times degradation may proceed at different rates because the absorption spectrum of degraded material has shifted. In Fig. S16 we overlay the initial change in XRD mean and PL mean for $FA_{0.83}Cs_{0.17}Pb(I_{0.7}Br_{0.3})_3$ at 85°C under both 470 nm and 590 nm. It can be seen that early time degradation dynamics are identical within error, demonstrating that our approach is valid. At subsequent times, degradation proceeds in a qualitatively similar manner but with differing rates because the absorption spectrum of the degraded structure has changed, leading to different absorbed photon flux for the different wavelengths (Figs. S2 and S8). Importantly, we chose to use 470 nm excitation in the majority of our measurements as sample absorption changes significantly less here than at longer wavelengths between pristine and degraded samples (Fig. S20) and we avoid any potential UV excitation or degradation effects[10].

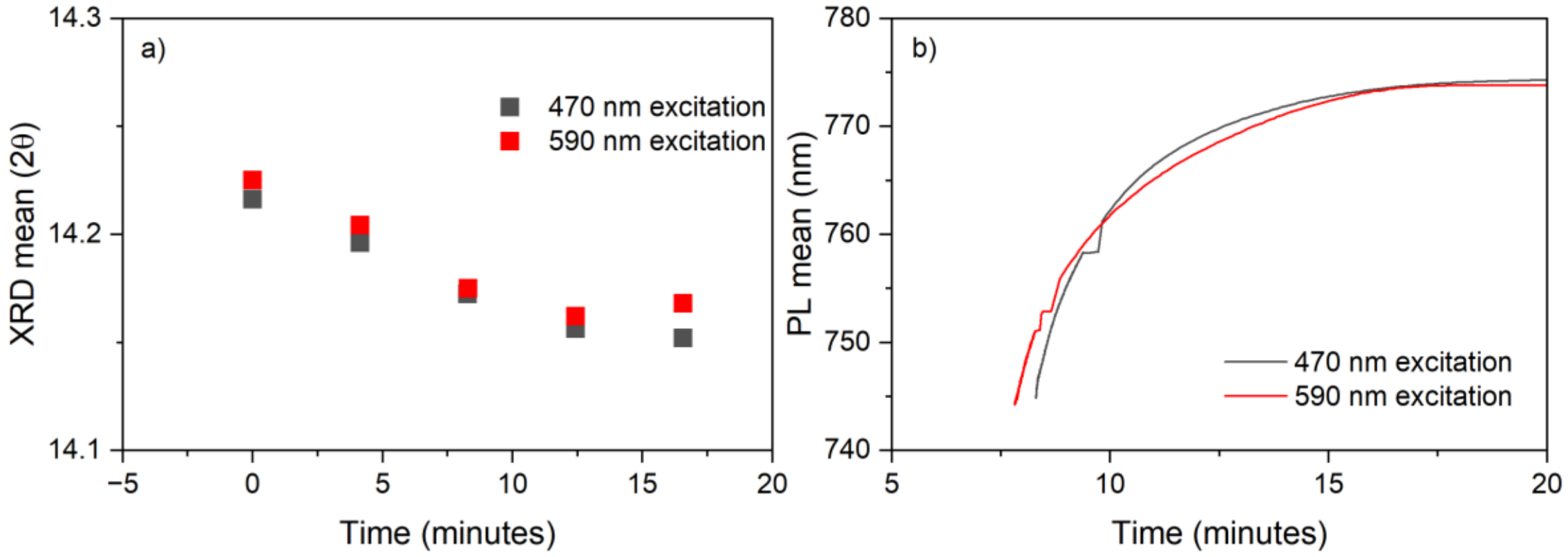

Fig. S16. Overlay of the early time degradation dynamics of a) mean XRD and b) photoluminescence mean of $FA_{0.83}Cs_{0.17}Pb(I_{0.7}Br_{0.3})_3$ when exciting at 470 nm and 590 nm. Photoluminescence data starts at minute 8 as this is when the heating stage reached temperature, as is noted in the methods section.

Supplementary Information Note 4 – further discussion on interpretation of main XRD and PL results

Here we discuss our interpretation of the XRD and PL dynamics in more detail. The mechanisms identified in the main text as contributors to degradation are: halide segregation (for $FA_{0.83}Cs_{0.17}Pb(I_{0.7}Br_{0.3})_3$), A-cation redistribution and finally structural fragmentation. These conclusions were reached from a holistic interpretation of all data available, rather than looking at any one data set individually. We comment on each of these processes in turn:

i) Halide segregation

Very similar changes to those observed in this study for both PL and XRD have been observed by others, meaning that our interpretation is well supported across the literature[9,11]. No other processes would concurrently lead to both a rapid shift in PL to lower energy, an increase in PL efficiency and broadening of XRD (Fig. 3b), all of which have been reported as individual hallmarks of halide segregation and are found here to occur together. Other likely processes can be ruled out when considering the complete data set: point defect formation would result in a significant drop in photoluminescence intensity and minimal change in the XRD, while local changes to dielectric functions or similar would be highly unlikely to cause such a significant change in the wavelength and would not cause any changes to the XRD spectra.

ii) A-cation redistribution

Considering $FA_{0.83}Cs_{0.17}Pb(I_{0.7}Br_{0.3})_3$ films first, the data presented in Fig. 3 of the main manuscript show that between 2 and 4 hours of light and heat exposure the XRD peak changes from a broad split peak to a single peak with reduced peak width, suggestive of strain relaxation. However, importantly, while the PL peak (i.e. mode) returns close to its initial wavelength, it is significantly broader than at time 0 and very asymmetric (as is shown by the peak width and skew in Fig. S2). We note that the cause cannot be a phase transformation – we monitored a wide range of 2θ angles (see Methods), especially regions where perovskite peaks exist, and saw no emergence of any new diffraction peaks. The PL peak being significantly broader and skewed when compared to time 0 also means that iodine and bromine have not returned to their original positions - remixing alone would not cause the PL to exhibit the observed asymmetry and broadening. We therefore propose that the most likely process that causes a uniform lattice spacing but a skewed PL peak is the motion of A-site cations (potentially alongside some halide motion), producing a uniform lattice spacing but varying bandgap across the sample. Our suggestion is motivated by comparable observations in the literature[12,13]. We suggest that the continued change to PL at longer times (without significant further changes to XRD) is explained by a reduction in charge-carrier funnelling to iodine rich domains. Funnelling is likely suppressed by defect formation, which captures charge carriers before they can reach iodide-rich domains, degradation of iodine rich

domains and potentially fracturing and isolation of material regions, as is supported by SEM images of the final structure having significant cracks across the surface.

For $FA_{0.83}Cs_{0.17}PbI_3$, the main change over a comparable timescale (< 6 hours) is a skewed photoluminescence peak becoming a more symmetric single peak (i.e. the mode of the PL peak wavelength is approaching the mean value, as shown in Figure 2g of the main manuscript). For the same reasons as those listed above we can again rule out phase transitions or defect evolution. We further rule out disorder or distortion effects given that the XRD peak width is unchanged (Fig. S11). Given that at the end of this time window a symmetric PL peak is observed (as shown by the skew approaching 0 in Fig. S11), this strongly suggests the formation of a more uniform electronic structure. This is most likely caused by more uniform mixing of FA and Cs (other potential processes would lead to a significant drop in PL intensity). This is comparable to the effect of annealing samples to improve optoelectronic quality[12].

iii) Iodine and FA loss

Our STEM-EDX results demonstrate that iodine is lost from the structure by the end of the measurement. Long-time changes are comparable in both structures (large reduction in XRD intensity and XRD peak moving to higher 2θ), both of which agree strongly with iodine loss. While STEM-EDX is not sensitive to FA, for $FA_{0.83}Cs_{0.17}PbI_3$ the shift in PL to such high energies cannot easily be explained by iodine loss alone. Specifically, the PL is given by the remaining structure and $FA_{0.83}Cs_{0.17}PbI_3$ does not have a bandgap sufficiently high in energy to produce this. While nanocrystals could in theory produce a sufficiently high energy bandgap, we would not expect a rapid jump in the PL peak (see Fig. 3c). Rather, the jump in PL mode is suggestive of a miscibility gap and, furthermore, Cs-majority halide perovskites have a bandgap at the observed PL energy[14]. Finally, for $FA_{0.83}Cs_{0.17}Pb(I_{0.7}Br_{0.3})_3$ we do not have definitive evidence that FA is lost from the structure. However, given both that $FA_{0.83}Cs_{0.17}PbI_3$ loses FA and that several literature reports suggest FA loss under varying conditions[15–17], our results are fully consistent with FA loss from the absorber layer[18].

Supplementary Information Note 5 – comparison of PL dynamics arising from halide segregation at different temperatures

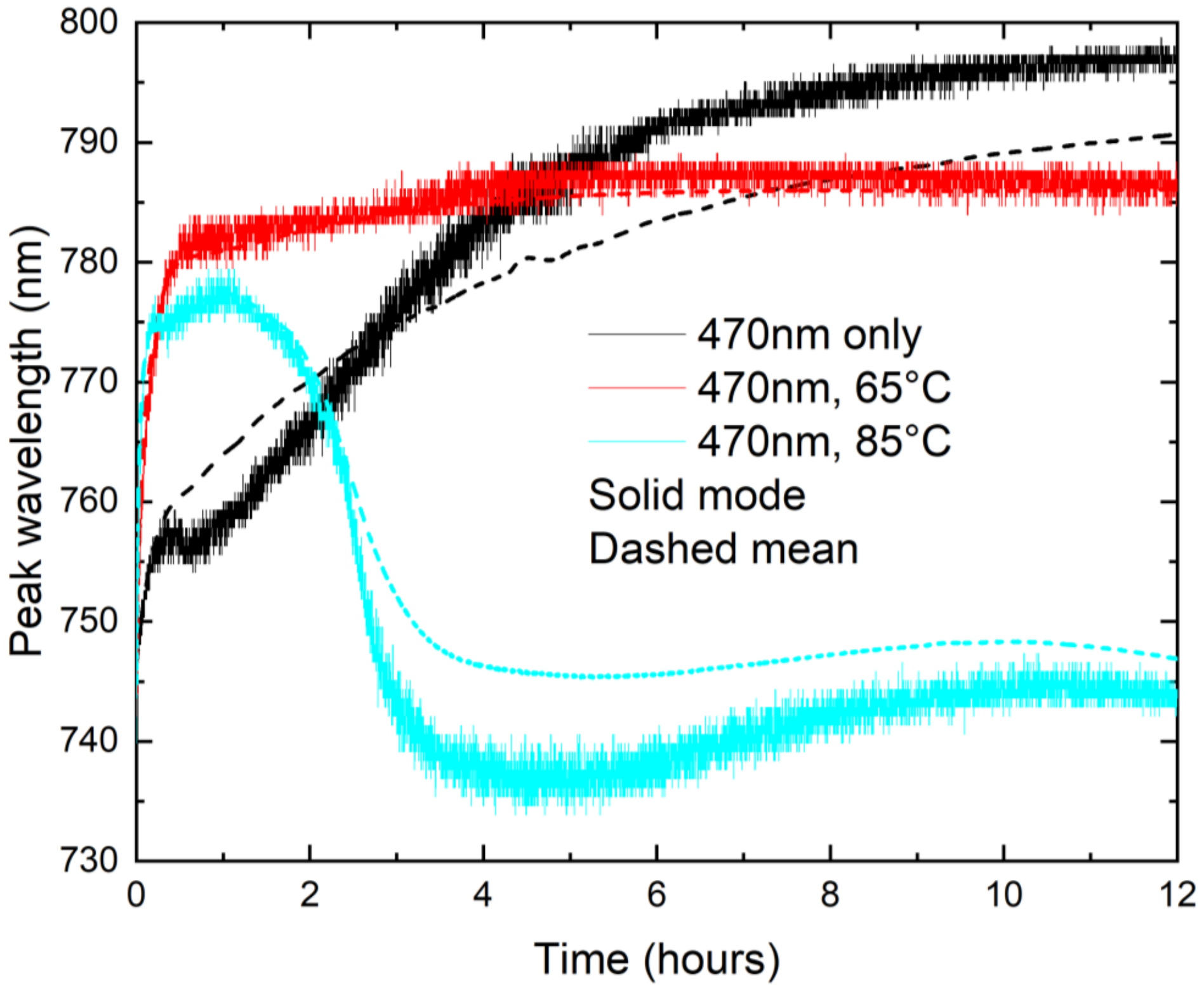

Fig. S17. Mode and mean photoluminescence peak for $FA_{0.83}Cs_{0.17}Pb(I_{0.7}Br_{0.3})_3$ film as a function of time, for illumination with 470-nm 1-sun equivalent light, while the material is held at either room temperature (22°C), 65°C or 85°C. It can be seen that the initial halide segregation dynamic is fastest at 85°C.

Supplementary Information Note 6 – XRD and PL spectra of a $FA_{0.83}Cs_{0.17}Pb(I_{0.7}Br_{0.3})_3$ film around the ~2 hour transition

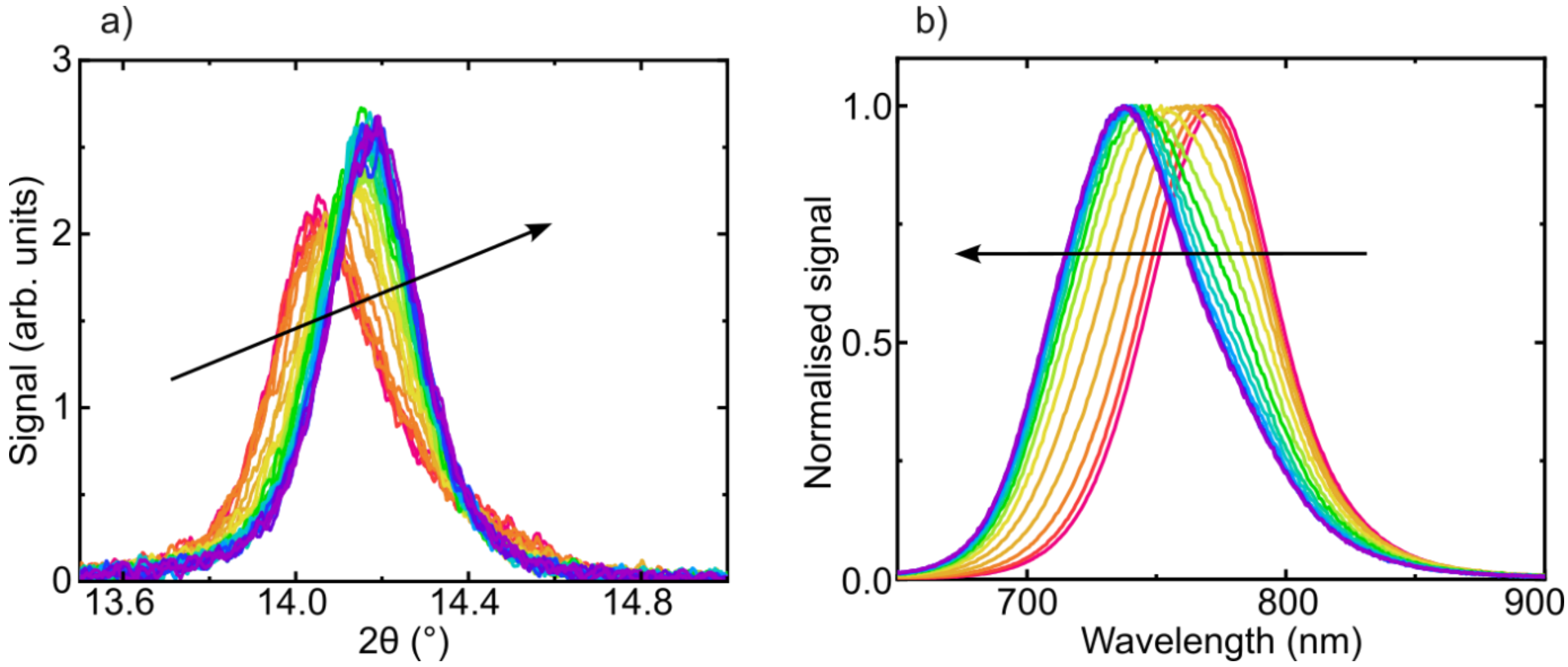

Fig. S18. a) XRD and b) photoluminescence spectra for a film of $FA_{0.83}Cs_{0.17}Pb(I_{0.7}Br_{0.3})_3$ when held at 85°C and illuminated with 1-sun at 470 nm. The data shown are between 1 hour 40 minutes and 2 hours 30 minutes on the time axis shown in Main Text Fig. 2. Arrows indicate direction of change. Red curve corresponds to the initial signal and purple the final signal. Time intervals between plots are constant.

Supplementary Information Note 7 – modelling degradation dynamics via XRD intensity decay

Here we discuss in more detail models designed to replicate the degradation rates inferred from XRD intensity decline under stressors. In Fig. S19a and b we plot the negative of the first derivative in XRD intensity, I_{XRD},versus time (t), i.e. $-\frac{dI_{\mathrm{XRD}}}{dt}$, where for notational simplicity we define $\frac{dI_{\mathrm{XRD}}}{dt} \equiv \dot{I_{\mathrm{XRD}}}$, for $FA_{0.83}Cs_{0.17}Pb(I_{0.7}Br_{0.3})_3$ and $FA_{0.83}Cs_{0.17}PbI_3$ respectively. The negative derivative $-\dot{I_{\mathrm{XRD}}}$ represents the time-dependent rate of degradation, as reflected by XRD intensity decline. Following early time effects (discussed below), as degradation progresses, the degradation rate $-\dot{I_{\mathrm{XRD}}}$ increases to a maximum for both $FA_{0.83}Cs_{0.17}Pb(I_{0.7}Br_{0.3})_3$ (Fig. S19a) and $FA_{0.83}Cs_{0.17}PbI_3$ (Fig. S19b) before returning towards 0. This observation underlines that the degradation rate is not constant with time.

We explored several models to fit such sigmoidal decay of the XRD intensity. Importantly, for $FA_{0.83}Cs_{0.17}Pb(I_{0.7}Br_{0.3})_3$ an initial faster reduction in I_{XRD} is observed, which stabilises within two hours, as can be seen in Fig. S19c. This initial decay corresponds to the period during which halide segregation occurs within the perovskite. As the decay kinetics are markedly different in this initial period compared with the subsequent slower material degradation, we omit this initial temporal region for fitting of the degradation dynamics in $FA_{0.83}Cs_{0.17}Pb(I_{0.7}Br_{0.3})_3$ based on either of the models presented below. For $FA_{0.83}Cs_{0.17}PbI_3$ the fits corresponding to the two approaches presented below represent the data well, but diverge slightly from the data at long times. Specifically, both fits approach 0 while the I_{XRD} value remains non-zero. This discrepancy is caused by the formation of a Cs-rich phase (as discussed in the main text), which gives a second contribution to the I_{XRD} in the same angular region. Two models that were successful in reproducing our data are the Autocatalytic and Avrami models[19,20], which we discuss in detail below. Results are presented in Table S1 both for fits to the thin films presented in the main text (referred to as sample 1, with data and fits presented in Figs. S19c and d) and for a second thin film of both compositions (referred to as sample 2). For $FA_{0.83}Cs_{0.17}Pb(I_{0.7}Br_{0.3})_3$ the data for the second thin film is presented in Fig. S9 (with the data recorded when heat and light had been turned off removed from the fit region), while for $FA_{0.83}Cs_{0.17}PbI_3$ the data for the second thin film are presented in Fig. S12. Fits are presented on these figures respectively. For both samples all values (and most importantly the rates extracted) agree reasonably well (noting some sample-to-sample variation is always anticipated).

Autocatalytic model

This model is based on the rate equation $A + B \leftrightharpoons 2B$[19], where in our case A represents the undegraded perovskite, whose remaining concentration is measured by I_{XRD}, and B the degraded material (which

we do not measure in our approach). Under the assumption that degraded material cannot reform into pristine material we can state

$$\dot{I}_{XRD} = -kI_{\mathrm{XRD}}(1 - bI_{\mathrm{XRD}}). \tag{1}$$

Here k and b are constants to be fitted and $(1 - bI_{\mathrm{XRD}})$ represents the concentration of degraded material. This equation has the solution

$$I_{\mathrm{XRD}} = \frac{1}{b + Ae^{kt}}. \tag{2}$$

We present the fit to I_{XRD} for both perovskites in Fig. S19 c and d, with extracted fitting parameters presented in Table S1.

Returning to equation 1, given that I_{XRD} is proportional to the total material present, we note it is possible to introduce the concept of the 'total fraction of pristine material', defined as $f_{\mathrm{M}} = bI_{\mathrm{XRD}}$, giving $\dot{f}_{\mathrm{M}} = -\frac{k}{b} f_{\mathrm{M}}(1 - f_{\mathrm{M}})$. This equation reflects that material loss is governed by the product of the pristine (f_{M}) and degraded $(1 - f_{\mathrm{M}})$ material fractions, multiplied by a fitting constant. One possible explanation for this formula is that degradation is driven by the interaction area (e.g. grain boundaries) between these two regions (assuming they are randomly distributed across the structure).

Avrami model

A second model we considered for fitting the recorded XRD intensity dynamics is the Avrami model[20]. This model describes the growth of crystalline domains, assuming they form randomly within the transforming volume and that each domain's growth rate is constant. The model also accounts for overlap of regions as they grow. The Avrami equation is

$$f = f_{\mathrm{max}}\left(1 - e^{-\left(\frac{t}{\tau}\right)^{c}}\right) \tag{3}$$

where f_{max} is the final fraction of crystalline material and τ and c are fitting constants. While this describes growth of a crystalline domain and that f is measurable, in the present scenario, this model would need to capture the growth of degraded domains while we measure the fraction of undegraded material. Thus for our system a reasonable variant on this model is

$$I_{\mathrm{XRD}} = Be^{-\left(\frac{t}{\tau}\right)^{\beta}}, \tag{4}$$

a form of stretched exponential with B a third fitting constant. We present fits to this model in Fig. S19 c and d and again find good agreement, with fit parameters presented in Table S1.

Notably, the assumption of constant growth rate relies on the degraded regions being in constant contact with pristine regions. Thus, while the rate of change with respect to I_{XRD} is significantly more complex in this model, the underlying assumptions again suggest an interaction between pristine and degraded regions.

Overall any model that describes a sigmoidal decay is likely to fit our data reasonably well. By definition any such model will have the property that $\frac{dI_{\mathrm{XRD}}}{dt}$ will tend towards 0 both when $I_{\mathrm{XRD}} \longrightarrow 0$ and when $(1 - DI_{\mathrm{XRD}}) \rightarrow 0$ (i.e. very little change will take place when only pristine or only degraded material is present). These models will therefore have the largest rate of change in the central portion of the decay. This finding strongly suggests that, independent of the exact model used, degradation is governed by interactions between pristine and degraded regions. While other possibilities exist, the simplest way for this to occur is at the regions where pristine and degraded material are in contact, for example at grain boundaries.

In the main text we present fits from the Autocatalytic model. While both approaches demonstrated above produce good fits, the assumption in the Avrami model of constant growth rate for degraded regions seems less likely to apply in the situation investigated here. Specifically, as the material degrades, fracturing occurs, meaning that the growth rate of degraded domains is likely to be affected by degradation processes that have already occurred.

	$FA_{0.83}Cs_{0.17}Pb(I_{0.7}Br_{0.3})_3$		$FA_{0.83}Cs_{0.17}PbI_3$	
Sample	1	2	1	2
**Autocatalytic model**				
b (arb. units)	0.833 ± 0.002	0.943 ± 0.003	0.971 ± 0.009	0.872 ± 0.023
A (arb. units)	0.0046 ± 0.0002	0.0048 ± 0.0003	0.155 ± 0.007	0.163 ± 0.019
k (hours^{-1})	0.369 ± 0.003	0.330 ± 0.003	0.364 ± 0.007	0.321 ± 0.009
**Avrami model**				
B (arb. units)	1.18 ± 0.01	1.051 ± 0.003	0.91 ± 0.01	0.94 ± 0.01
τ (hours)	15.71 ± 0.02	17.83 ± 0.03	7.91 ± 0.05	7.82 ± 0.08
β	3.70 ± 0.03	3.68 ± 0.04	1.86 ± 0.03	1.68 ± 0.04

Table S1. Fitting parameters for the fits discussed in this supplementary note and presented in Fig. S19 for samples 1, and Figs. S9 and S12 for samples 2. Errors represent standard deviation in fitting values.

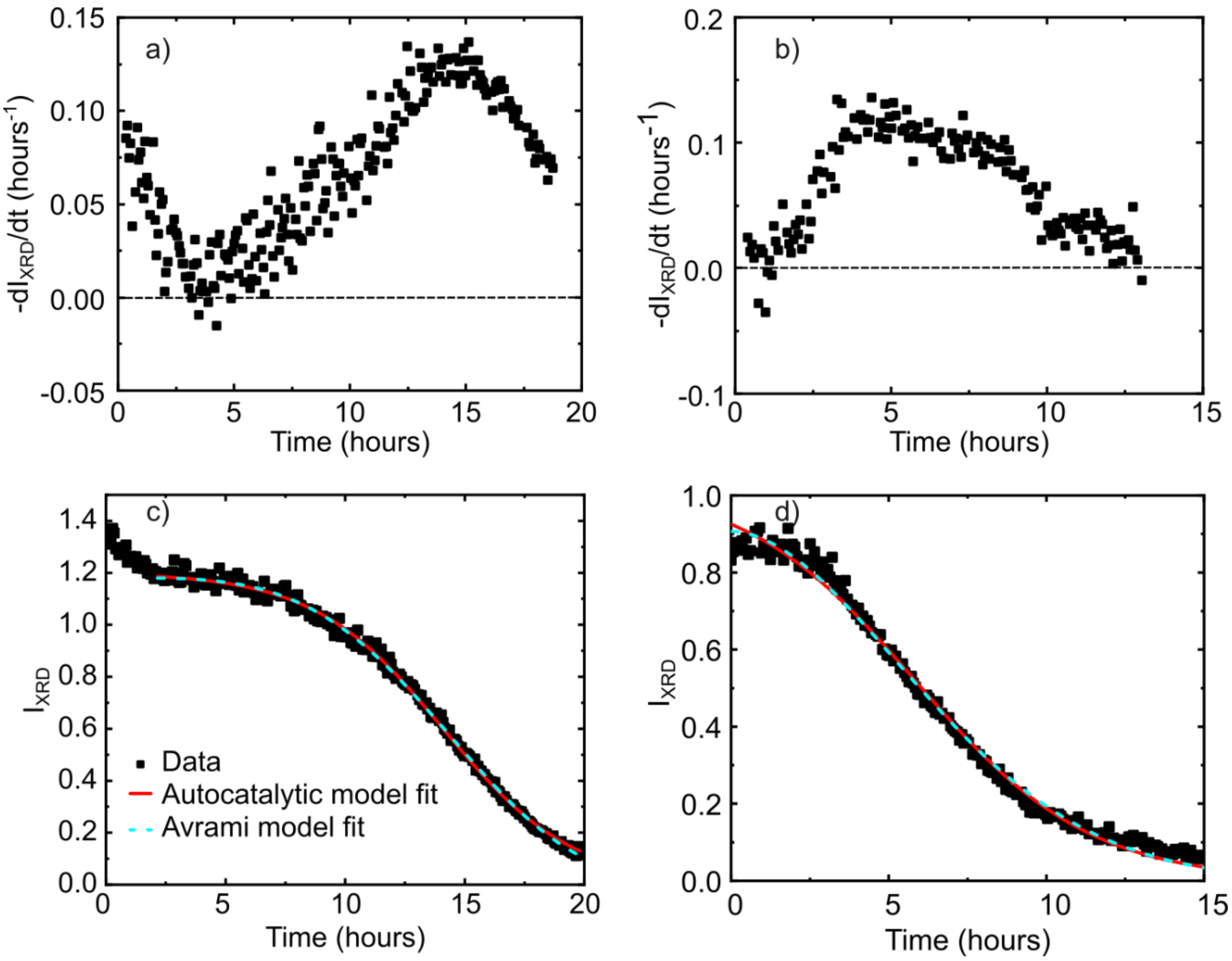

Fig. S19. The degradation rate, $-\dot{I}_{\mathrm{XRD}}$, versus time for a) $FA_{0.83}Cs_{0.17}Pb(I_{0.7}Br_{0.3})_3$ and b) $FA_{0.83}Cs_{0.17}PbI_3$ respectively. 20-point adjacent averaging has been applied to I_{XRD} prior to calculating the derivative. Dashed line marks 0. Fits to data (i.e. without adjacent averaging) with both the Autocatalytic and Avrami model, as described in the Supplementary Information, for c) $FA_{0.83}Cs_{0.17}Pb(I_{0.7}Br_{0.3})_3$ and d) $FA_{0.83}Cs_{0.17}PbI_3$ respectively. For $FA_{0.83}Cs_{0.17}PbI_3$ we only present and fit the region where we are confident of no contribution to the XRD signal from a Cs-rich phase (i.e. below 15 hours). Legend in c) applies to c) and d).

Supplementary Information Note 8 – absorption measurements

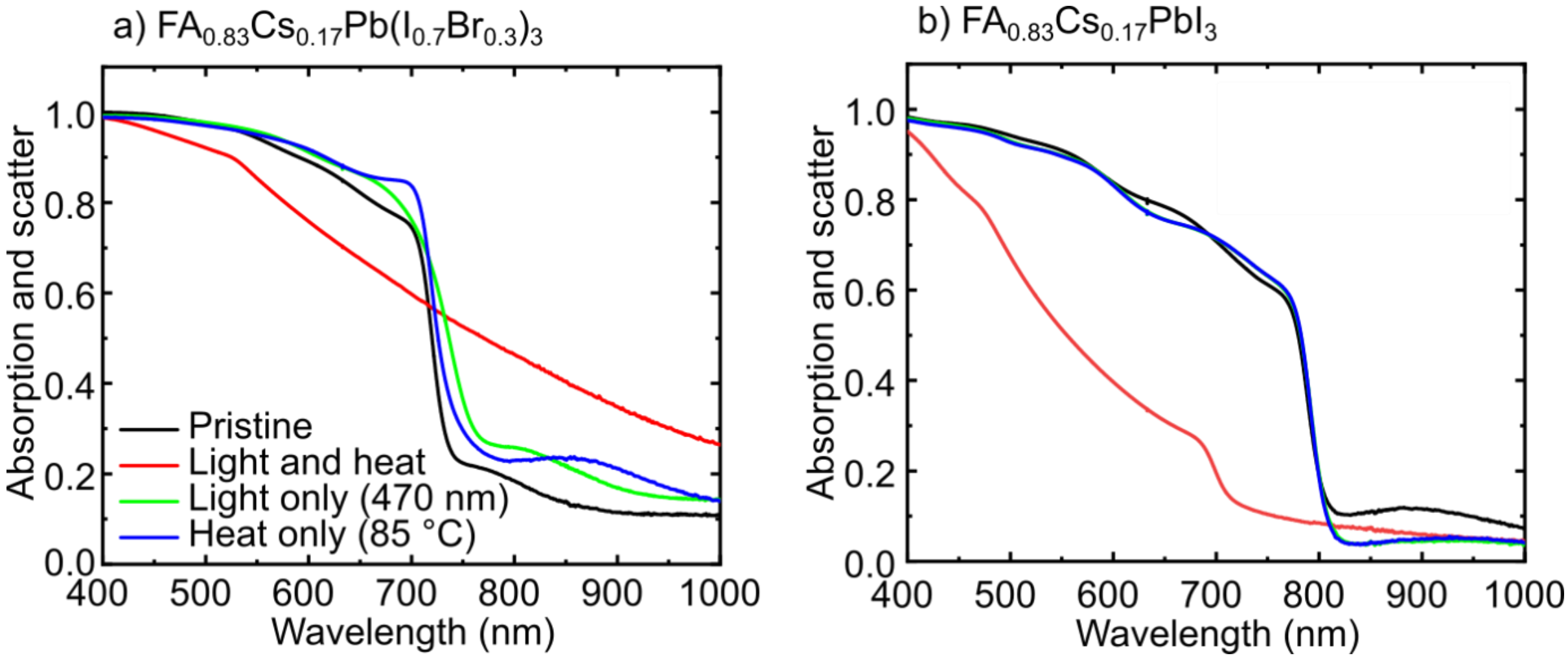

Fig. S20. Absorption and scatter (i.e. 1-Transmission-Reflection) measurements of a) $FA_{0.83}Cs_{0.17}Pb(I_{0.7}Br_{0.3})_3$ and b) $FA_{0.83}Cs_{0.17}PbI_3$ for pristine (fresh) and degraded samples, as noted in legend (for 20 hours of exposure). Total exposure of samples to light was less than 3 minutes per sample. Legend in a) applies to both plots.

Supplementary Information Note 9 – optical images of all samples

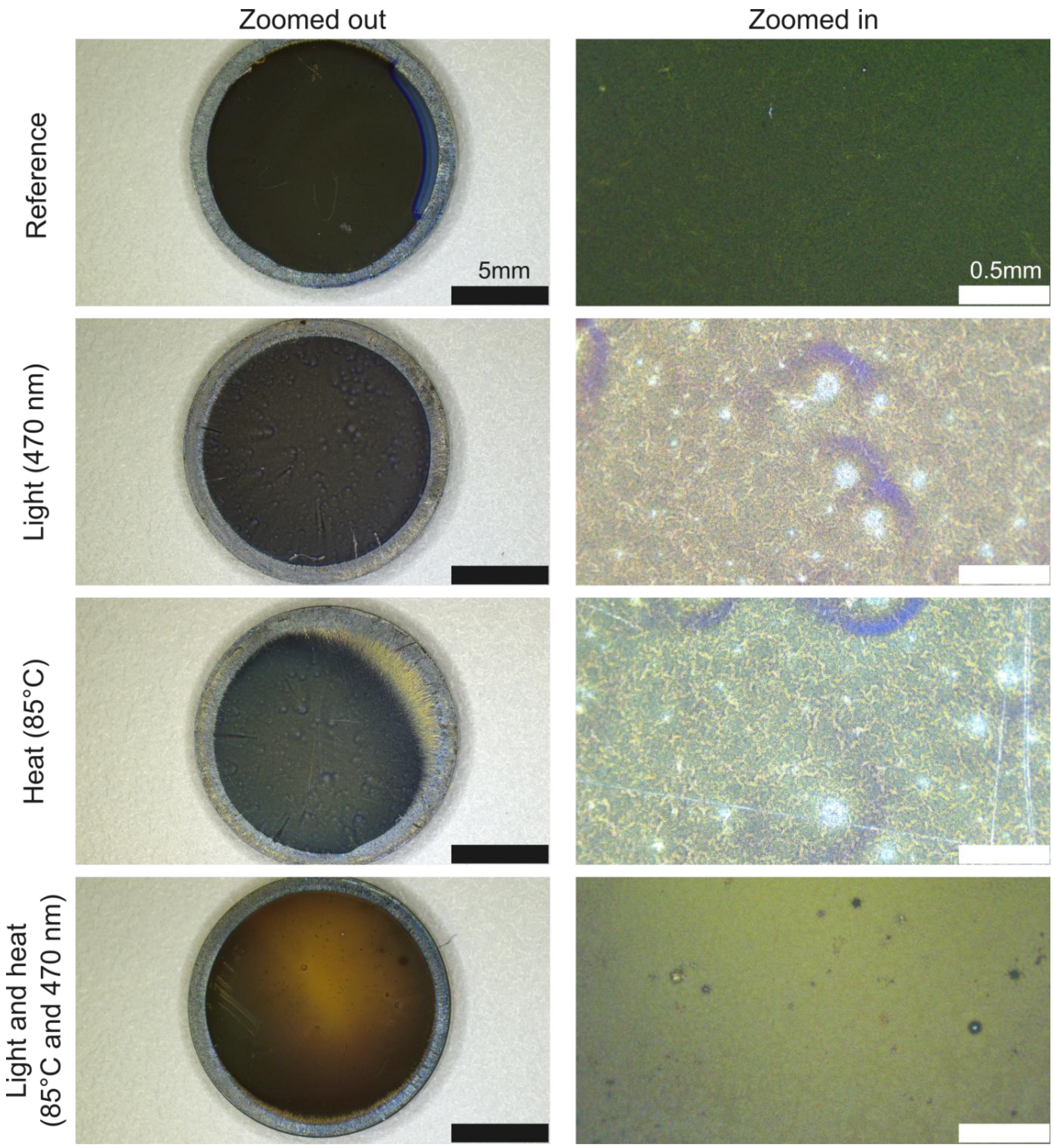

Fig. S21. Optical images of $FA_{0.83}Cs_{0.17}Pb(I_{0.7}Br_{0.3})_3$ films on quartz substrates, before (reference) and after degradation for 20 hours under the conditions stated. We note that an auto-exposure setting was used, allowing better observation of samples when zoomed in, but that the colours observed here are not representative of what would be seen with the naked eye (left hand images are comparable to what is observed by eye).

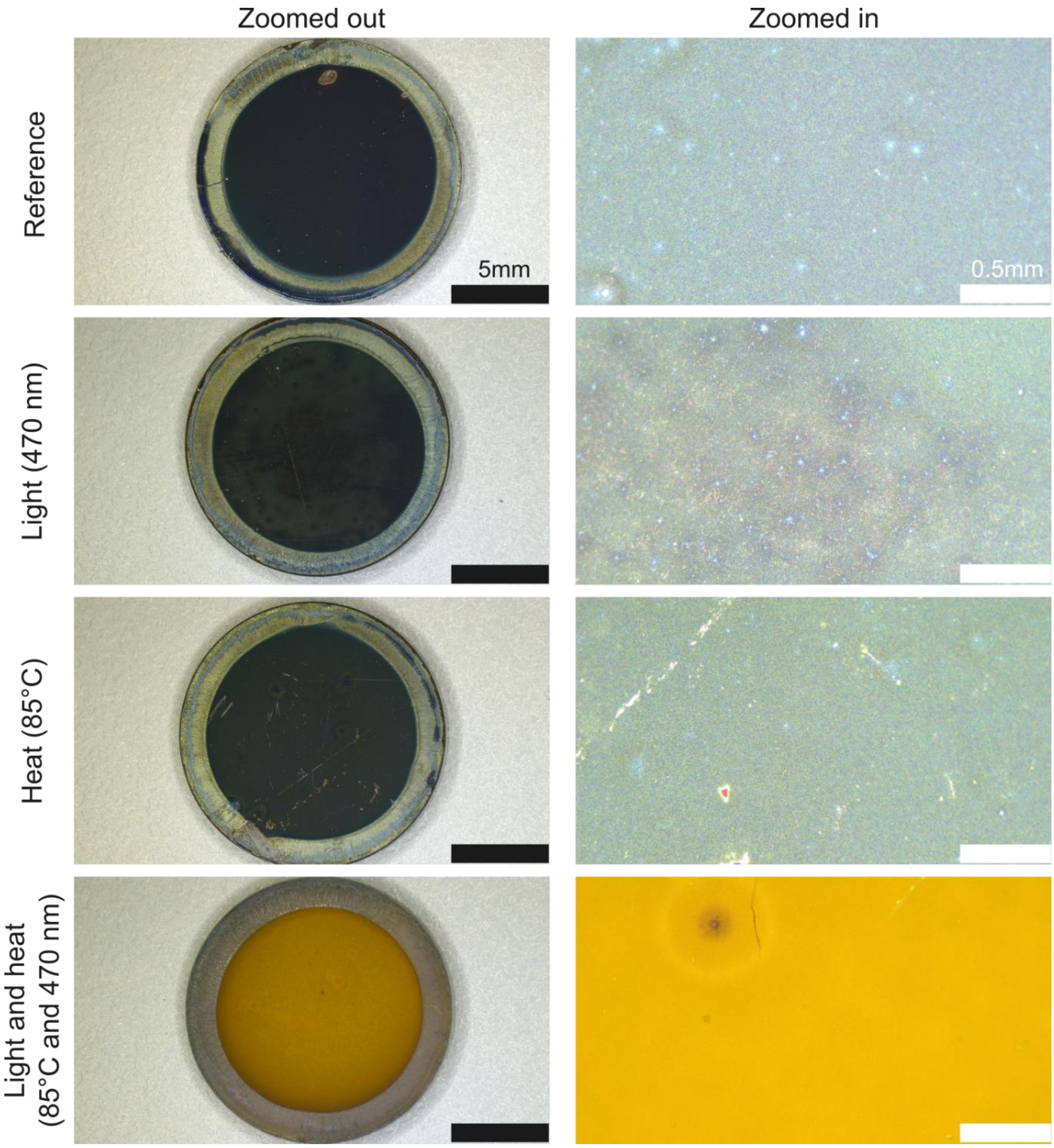

Fig. S22. Optical images of $FA_{0.83}Cs_{0.17}PbI_3$ films on quartz substrates, before and after degradation for 20 hours under the conditions stated. We note that an auto-exposure setting was used, allowing better observation of samples when zoomed in, but that the colours observed here are not representative of what would be seen with the naked eye (left hand images are comparable to what is observed by eye).

Supplementary Information Note 10 – additional SEM images of degraded sample

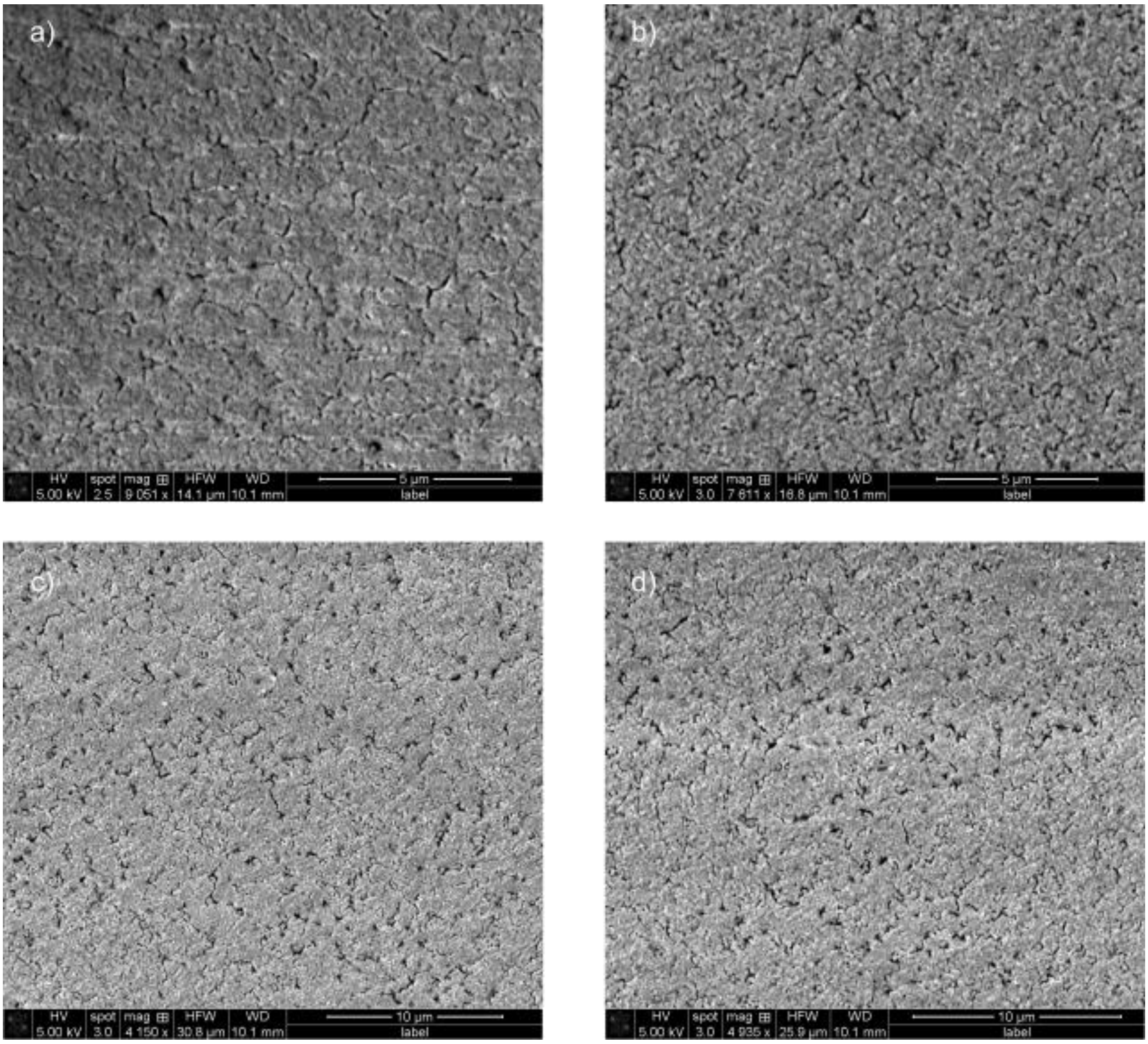

Fig. S23. a) – d) present additional SEM images of nominal $FA_{0.83}Cs_{0.17}Pb(I_{0.7}Br_{0.3})_3$ films following exposure to 20 hours of heat (85°C) and light (1-sun equivalent, 470 nm). As noted in the main text, small charging artefacts can be seen on all images due to the degraded nature of the films. Samples were washed in a chlorobenzene bath to remove the PMMA encapsulant prior to SEM images being recorded.

Supplementary Information Note 11 – steady state and time-resolved TRPL overlay

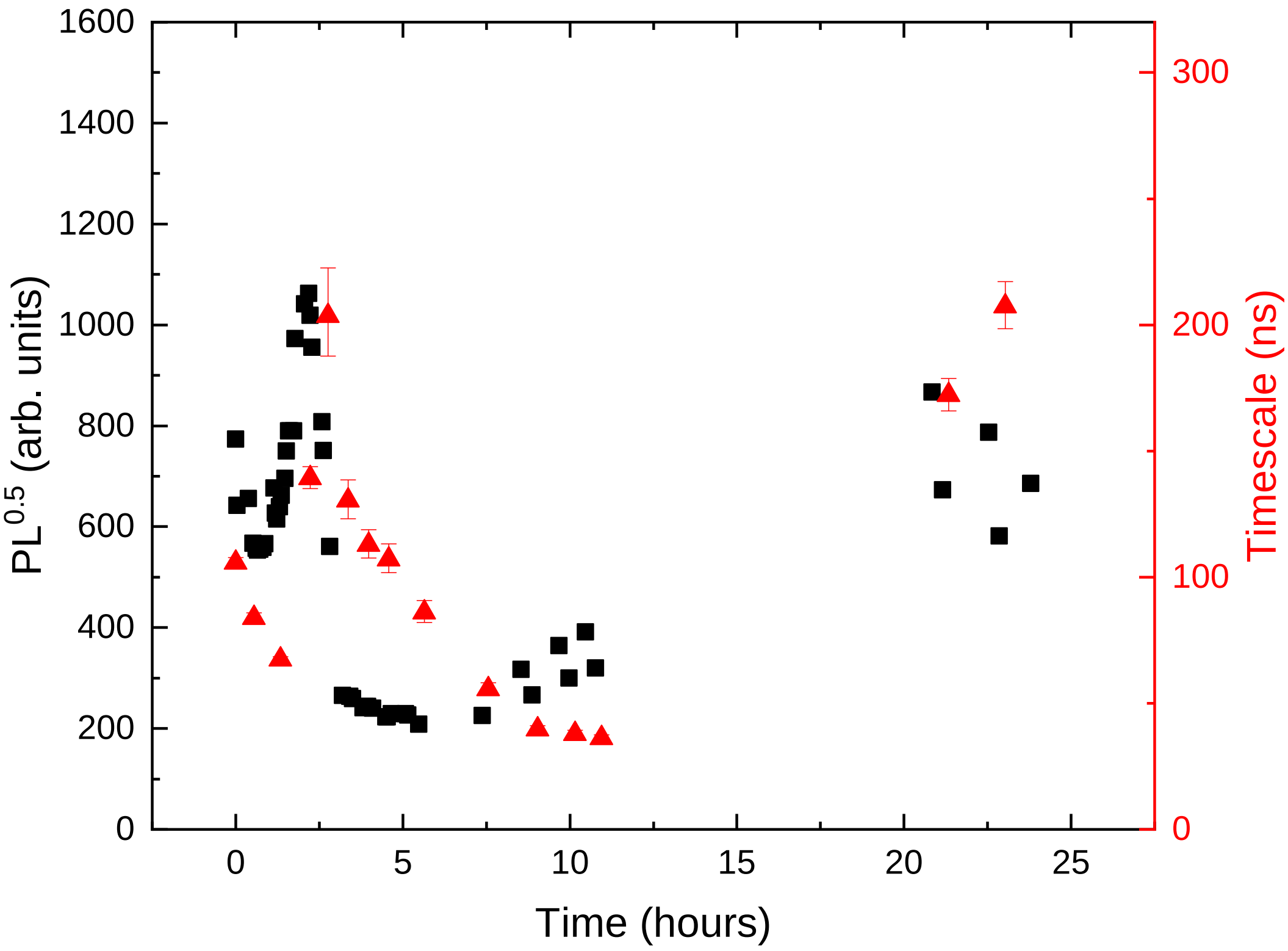

Fig. S24. Overlay of the square root of photoluminescence and decay timescales for $FA_{0.83}Cs_{0.17}Pb(I_{0.7}Br_{0.3})_3$ when held at 85°C and illuminated with 1-sun at 470 nm.

In Fig. S24 we overlay TRPL lifetimes with the square root of the continuous-wave PL intensity, measured on the same experimental setup. Specifically, we found that TRPL transients followed first-order decays after the first ~10 ns, with the initial faster decay component attributed to a mix of second-order electron-hole recombination and charge redistribution within the material. Following this initial region, the charge-carrier density can therefore be described by $\frac{dn}{dt} \approx -\frac{n}{\tau_0}$, meaning $n \propto e^{-\frac{t}{\tau_0}}$. Therefore $TRPL \propto n^2 \propto e^{-\frac{2t}{\tau_0}}$. Similarly, for continuous-wave PL within a trap-dominated regime we can state $G \approx \frac{n}{\tau_0}$, where G is the local charge-carrier generation rate. Therefore $PL \propto n^2 \propto (G\tau_0)^2$. Therefore $\tau_0 \propto (PL)^{0.5}$, hence presenting these terms in Fig. S24 (noting τ_0 is denoted as timescale on the figure). Due to different excitation wavelengths (470 nm versus 405 nm for LED/TRPL) and spot sizes (see Methods) we do not anticipate perfect agreement between the two results, though in both cases we mainly recorded signal from the centre of the sample. The results in Fig. S24 demonstrate qualitatively similar trends in both datasets.

Supplementary Information Note 12 – time scaling and 2θ overlay

To carry out a global fit on our PL and XRD data we converted PL intensity, PL mean wavelength, XRD intensity and XRD mean wavelength data into dimensionless quantities with the following transform

$$\chi_i = \frac{x_i - \mu}{\sigma}, \tag{6}$$

where x_i represents individual data point i, μ the mean of the dataset and σ the standard deviation of the dataset. We then computed the following value

$$V = \frac{1}{N}\sum_{i}\Big(\left|\chi_{\mathrm{IPL,RT},i}(t) - \chi_{\mathrm{IPL,T},i}(\alpha t)\right| + \left|\chi_{\lambda\mathrm{PL,RT},i}(t) - \chi_{\lambda\mathrm{PL,T},i}(\alpha t)\right| + \left|\chi_{\mathrm{IXRD,RT},i}(t) - \chi_{\mathrm{IXRD,T},i}(\alpha t)\right| + \left|\chi_{2\theta,\mathrm{RT},i}(t) - \chi_{2\theta,\mathrm{T},i}(\alpha t)\right|\Big). \tag{7}$$

Here N is the total number of data points, IPL denotes PL intensity, λPL denotes PL mean wavelength, IXRD XRD intensity, 2θ mean XRD value, RT room temperature, T the elevated temperature of 85°C and α a time scaling constant. Note high temperature variables were interpolated to have the same number of data points in the time region studied as the room temperature data. This equation is a measure of the difference between high temperature and room temperature values as a function of scaled time αt. We varied α and calculated the equation above, which allowed us to obtain the best scaling value and minimum of the equation. Results are presented in Fig. S25. A minimum is found at 53, giving the scaling factor.

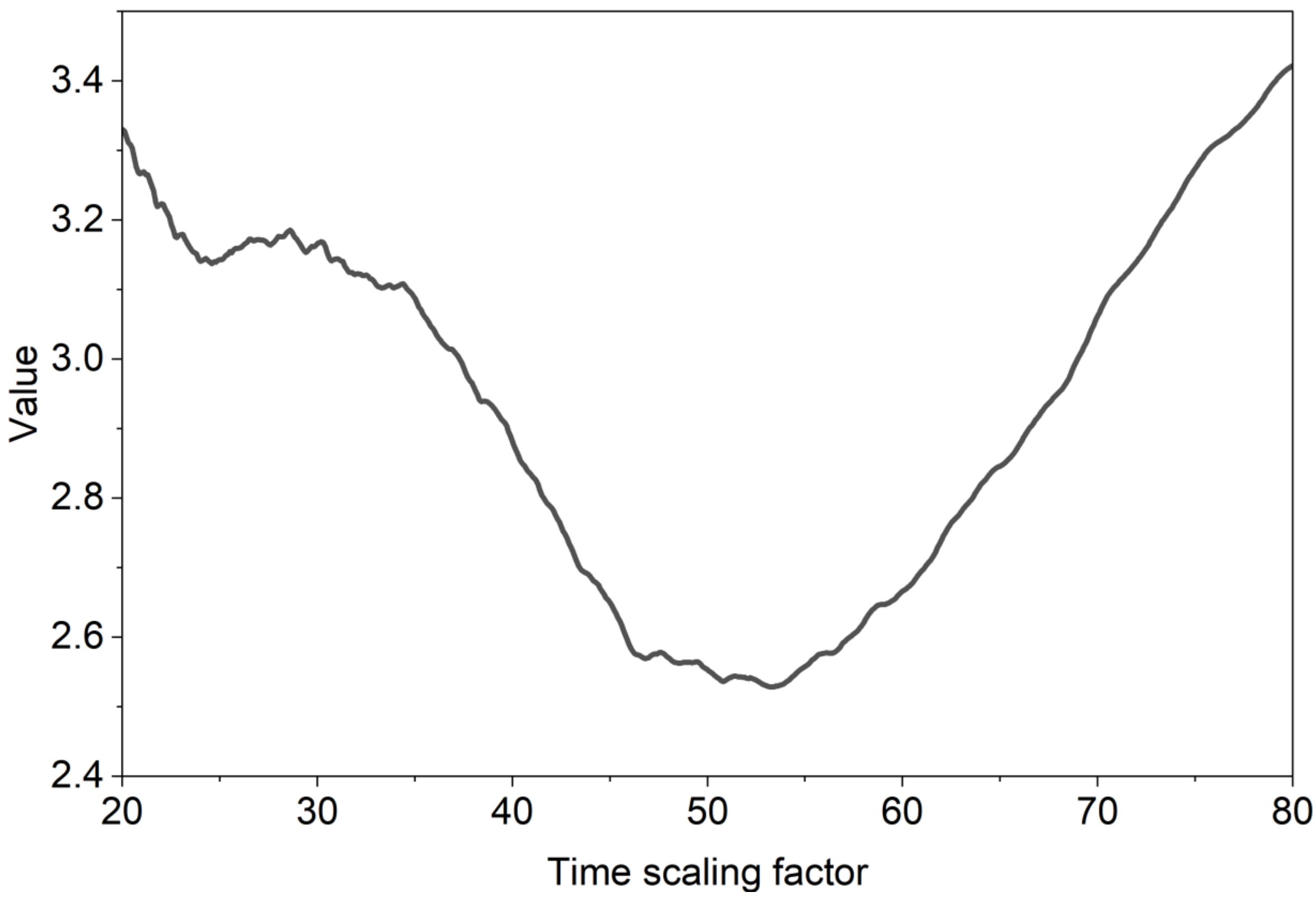

Fig. S25. Value of V as the time scaling factor, α, is varied. A minimum is obtained at 53.

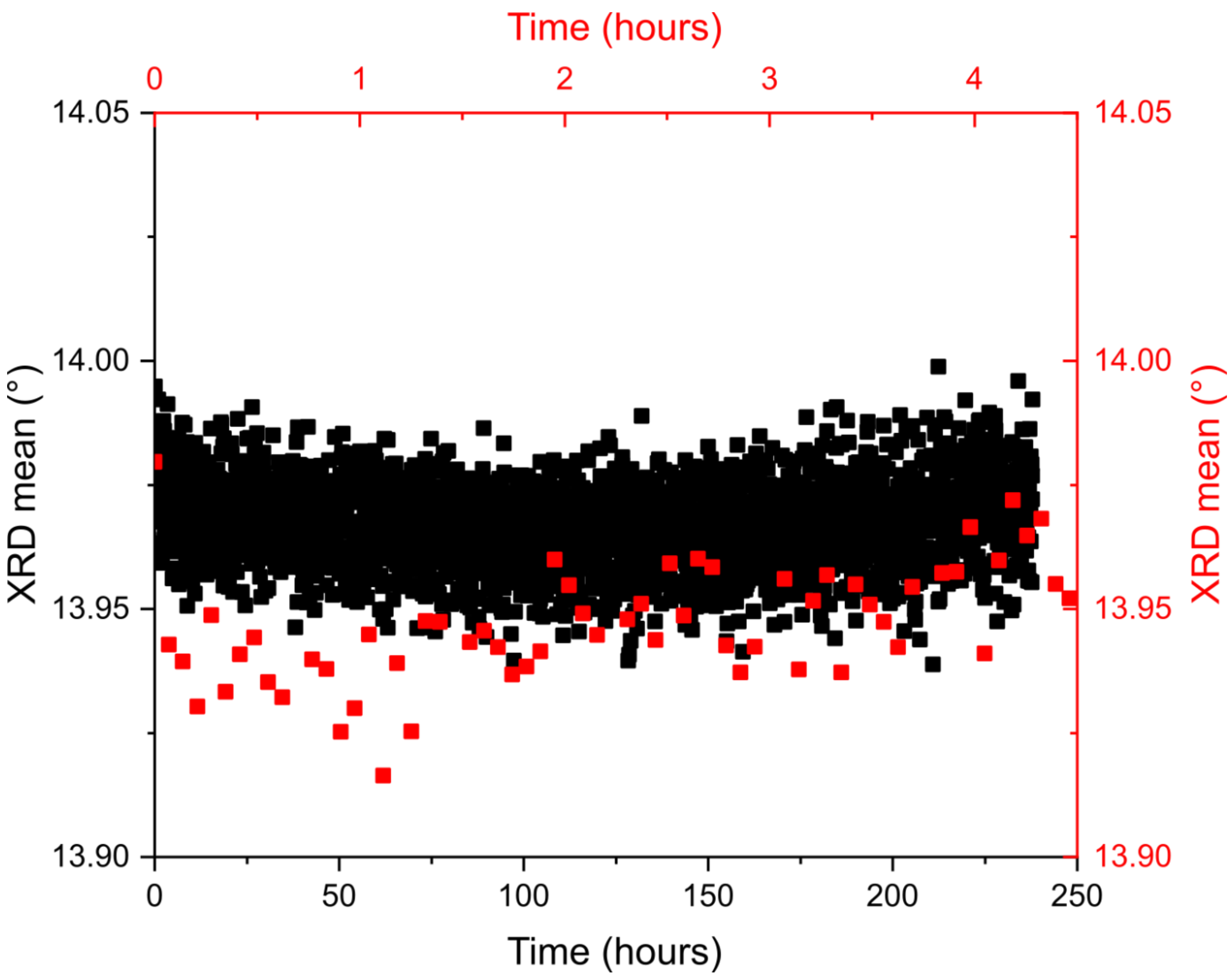

Fig. S26. XRD mean 2θ (in the range 10.4°-16.1°) overlaid for two $FA_{0.83}Cs_{0.17}PbI_3$ films, one exposed to 470 nm at room temperature (black lines and markers) and a second exposed to 85°C and 470 nm (red lines and markers). Note that the two datasets are presented with respect to different time axes for room temperature (bottom, black) and 85°C (top, red) with relative axes scaling (×53) chosen so that the functional form of the photoluminescence curves overlap. The shift in red XRD data following time 0 is due to thermal expansion.

Supplementary Information Note 13 – excitation power and typical illumination spot

Using our measurements of the LED spot size, we can write that its intensity in the thin-film plane as a function of x and y is given by $I(x, y) = I_0 f(x, y)$, where $f(x = 0, y = 0) = 1$ is the maximum value of the function. Therefore, I_0 represents the maximum intensity of the LED illumination spot. We found that the illumination intensity varies slowly over the illumination area and was much larger than the XRD illumination spot (see Fig. S27 for a plot of the typical LED illumination spot). We set I_0 to generate the same number of excitations as in 1-sun illumination, specifically

$$I_0 = \frac{\int a(\lambda)\phi_{\mathrm{AM1.5}}(\lambda)d\lambda}{a(\lambda_{\mathrm{LED}})} \times E_{\mathrm{LED}} \tag{8}$$

where a is the fraction of light absorbed by the sample at wavelength λ, $\phi_{\mathrm{AM1.5}}$ the (global) AM1.5 photon flux per unit wavelength, λ_{LED} the central wavelength of the LED and E_{LED} the energy of a photon at the LED wavelength. We calculated the corresponding power to be measured on a power meter (a Coherent Powermax PM10 power head and FieldMaxII-TO), given by $P = \int I_0 f(x, y)dxdy$ and measured the LED power at the sample location. Finally, we note (as can be seen in Supplementary Information Note 8) that our absorption measurements contain some below-bandgap scatter. To account for this we carried out a linear interpolation of the band edge to obtain an approximate bandgap, and set the below-bandgap absorption to 0. We note different methods for carrying out this interpolation result in variation of P of less than 5 %. The irradiance values calculated for different measurements are presented in Table S2. Finally, we estimate the total error in this approach to be less than 10 %.

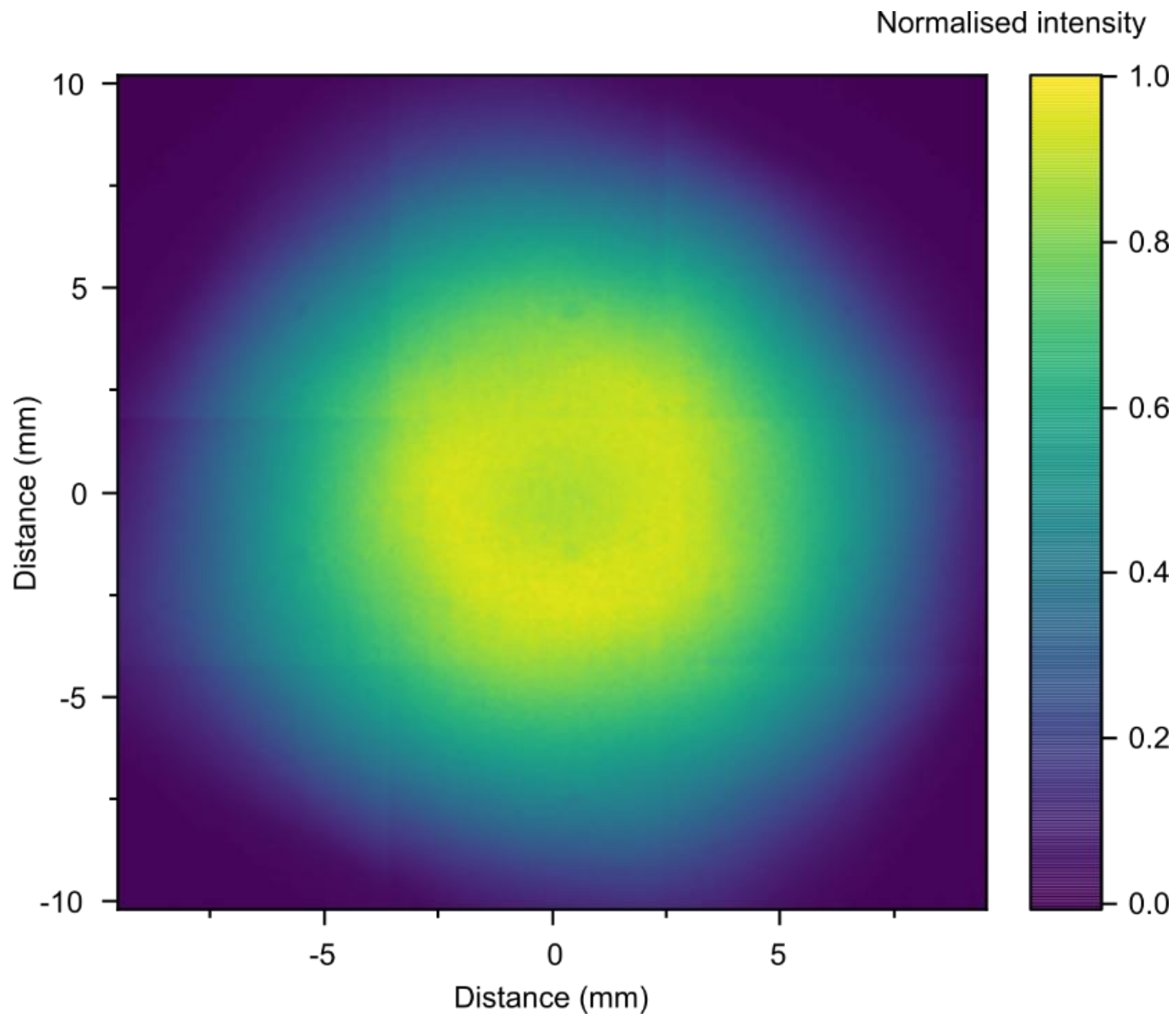

Fig. S27. Example of illumination spot produced by LED used for light-induced degradation experiments.

Measurement	*Incident LED intensity (mWcm^{-2})*
$FA_{0.83}Cs_{0.17}Pb(I_{0.7}Br_{0.3})_3$, 470 nm illumination	54 ± 3
$FA_{0.83}Cs_{0.17}Pb(I_{0.7}Br_{0.3})_3$, 590 nm illumination	47 ± 3
$FA_{0.83}Cs_{0.17}PbI_3$, 470 nm illumination	65 ± 4

Table S2. Incident laser intensities required for different measurements.

Supplementary Information Note 14 – raw EDX data

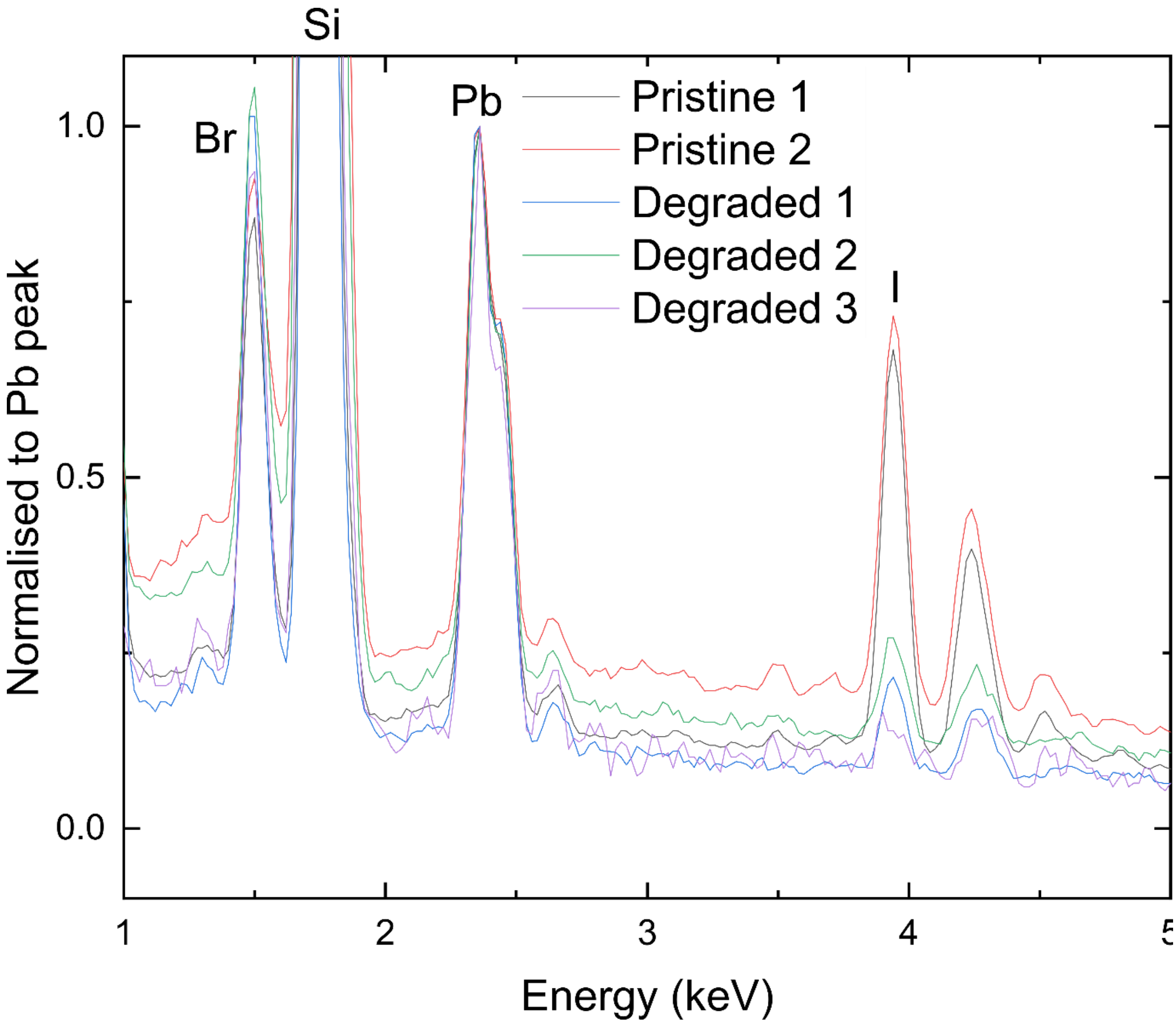

Fig. S28. Raw cross-sectional EDX data for measurements on pristine and degraded (20 hours 85°C and 1-sun 470 nm equivalent) $FA_{0.83}Cs_{0.17}Pb(I_{0.7}Br_{0.3})_3$ films. All data were normalised to the Pb peak maximum. Elements identified are noted on the plot. Different datasets relate to different spots across the film surface.

References

1. Eperon, G. E. *et al.* The Importance of Moisture in Hybrid Lead Halide Perovskite Thin Film Fabrication. *ACS Nano* **9**, 9380–9393 (2015).
2. Castriotta, L. A. *et al.* Challenges, technological pathways and trade-offs of perovskite solar modules for long-term operation. *Nat Energy* **11**, 534–546 (2026).
3. Ranka, A. *et al.* Cracking in polymer substrates for flexible electronic devices and its mitigation. *npj Flex Electron* **9**, 92 (2025).
4. Zhao, X. *et al.* Accelerated aging of all-inorganic, interface-stabilized perovskite solar cells. *Science* **377**, 307–310 (2022).
5. Jiang, Q. *et al.* Towards linking lab and field lifetimes of perovskite solar cells. *Nature* **623**, 313–318 (2023).
6. Haeger, T., Heiderhoff, R. & Riedl, T. Thermal properties of metal-halide perovskites. *Journal of Materials Chemistry C* **8**, 14289–14311 (2020).
7. Hu, Z., Aigouy, L., Chen, Z. & Fournier, D. Thermal conductivity and diffusivity of triple-cation perovskite halide materials for solar cells. *J. Appl. Phys.* **127**, 125113 (2020).
8. Zhang, X. R., Kong, G. Q., Chen, Y. H., Chen, L. & Chen, G. Measurement and Prediction of the Thermal Conductivity of Fused Quartz in the Range of 5–45 °C. *Int J Thermophys* **42**, 122 (2021).
9. Knight, A. J. *et al.* Halide Segregation in Mixed-Halide Perovskites: Influence of A-Site Cations. *ACS Energy Lett.* **6**, 799–808 (2021).
10. Chen, T., Xie, J. & Gao, P. Ultraviolet Photocatalytic Degradation of Perovskite Solar Cells: Progress, Challenges, and Strategies. *Advanced Energy and Sustainability Research* **3**, 2100218 (2022).
11. Halford, G. C. *et al.* Structural Dynamics of Metal Halide Perovskites during Photoinduced Halide Segregation. *ACS Appl. Mater. Interfaces* **14**, 4335–4343 (2022).

12. Mundt, L. E. *et al.* Mixing Matters: Nanoscale Heterogeneity and Stability in Metal Halide Perovskite Solar Cells. *ACS Energy Lett.* **7**, 471–480 (2022).

13. Ho, K., Wei, M., Sargent, E. H. & Walker, G. C. Grain Transformation and Degradation Mechanism of Formamidinium and Cesium Lead Iodide Perovskite under Humidity and Light. *ACS Energy Lett.* **6**, 934–940 (2021).

14. Xu, Z. *et al.* Phase diagram and stability of mixed-cation lead iodide perovskites: A theory and experiment combined study. *Phys. Rev. Mater.* **4**, 095401 (2020).

15. Wang, M. *et al.* Decoupling cation segregation and volatile loss in formamidinium–caesium metal halide perovskite solar cells under high-temperature operating conditions. *Nat Energy* **11**, 1–12 (2026).

16. Akbulatov, A. F. *et al.* Intrinsic thermal decomposition pathways of lead halide perovskites APbX3. *Solar Energy Materials and Solar Cells* **213**, 110559 (2020).

17. Kroll, M. *et al.* Insights into the evaporation behaviour of FAI: material degradation and consequences for perovskite solar cells. *Sustainable Energy Fuels* **6**, 3230–3239 (2022).

18. Emelianov, N. A. *et al.* Synergistic stabilization of lead halide perovskites by univalent cations under electric field stress. *Sustainable Energy & Fuels* **10**, 258–266 (2026).

19. Šimon, P. Fourty years of the Šesták–Berggren equation. *Thermochimica Acta* **520**, 156–157 (2011).

20. Porter, D. A., Easterling, K. E. & Sherif, M. Y. *Phase Transformations in Metals and Alloys*. (CRC Press, Boca Raton, 2021). doi:10.1201/9781003011804.
